# Titanium Dioxide Nanoparticles: Effects on Development and Male Reproductive System

**DOI:** 10.3390/nano13111783

**Published:** 2023-05-31

**Authors:** Elena Maria Scalisi, Roberta Pecoraro, Antonio Salvaggio, Fabiano Capparucci, Cosimo Gianluca Fortuna, Massimo Zimbone, Giuliana Impellizzeri, Maria Violetta Brundo

**Affiliations:** 1 Department of Biological, Geological and Environmental Science, University of Catania, 95124 Catania, Italy; roberta.pecoraro@unict.it (R.P.); mariavioletta.brundo@unict.it (M.V.B.); 2 Experimental Zooprophylactic Institute of Sicily “A. Mirri”, 90129 Palermo, Italy; antonio.salvaggio@izssicilia.it; 3 Department of Chemical, Biological, Pharmacological and Environmental Science, University of Messina, 98166 Messina, Italy; fcapparucci@unime.it; 4 Department of Chemical Sciences, University of Catania, 95124 Catania, Italy; cg.fortuna@unict.it; 5 CNR-IMM, 95123 Catania, Italy; massimo.zimbone@ct.infn.it (M.Z.); giuliana.impellizzeri@ct.infn.it (G.I.)

**Keywords:** TiO_2_-NPs, *Danio rerio*, male infertility, embryonic development, endocrine system, testis

## Abstract

Titanium dioxide nanoparticles (TiO_2_-NPs) are used intensively. Thanks to their extremely small size (1–100 nm), TiO_2_-NPs are more absorbable by living organisms; consequently, they can cross the circulatory system and then be distributed in various organs including the reproductive organs. We have evaluated the possible toxic effect of TiO_2_-NPs on embryonic development and the male reproductive system using *Danio rerio* as an organism model. TiO_2_-NPs (P25, Degussa) were tested at concentrations of 1 mg/L, 2 mg/L, and 4 mg/L. TiO_2_-NPs did not interfere with the embryonic development of *Danio rerio*, however, in the male gonads the TiO_2_-NPs caused an alteration of the morphological/structural organization. The immunofluorescence investigation showed positivity for biomarkers of oxidative stress and sex hormone binding globulin (SHBG), both confirmed by the results of qRT-PCR. In addition, an increased expression of the gene responsible for the conversion of testosterone to dihydrotestosterone was found. Since Leydig cells are mainly involved in this activity, an increase in gene activity can be explained by the ability of TiO_2_-NPs to act as endocrine disruptors, and, therefore, with androgenic activity.

## 1. Introduction

The technology-based industry realized that nanoparticles presented potential opportunities such as increased energy efficiency and cleaning up industrial contaminants [1]. The use of nanotechnology helps decrease production costs by reducing energy consumption, attenuating environmental pollution, and increasing production efficiencies in developed countries.

The manufacturing of nanotechnology are the engineered nanoparticles (ENPs) in which the rearrangement of the atoms gives them new properties [2,3]. Titanium dioxide nanoparticles (TiO_2_-NPs), especially, are among the most engineered metal oxide nanoparticles in the world [4,5] and are used to remove pollutants from wastewater [6]. Presently, their photocatalysis property is considered an efficient methodology in the area of wastewater treatment [7,8] because TiO_2_-NPs are minimally selective; therefore, many types of contaminants such as polycyclic aromatic hydrocarbons [9], chlorinated organic compounds [10], dyes [11], pesticides, cyanide [12], phenols [13], arsenic [14], and heavy metals [15] can be degraded by TiO_2_ nanoparticles. Moreover, TiO_2_-NPs are used in a wide variety of products, such as food colorants (under E code number E171), nutritional supplements, personal care products (cosmetics, sunscreens), toothpaste [16,17], and paint [18].

The increasing production and use of manufactured nanoparticles, such as titanium dioxide nanoparticles (TiO_2_-NPs), has inevitably led to their release into the aquatic environment; thereby posing a threat to aquatic organisms and humans alike [19,20]. Their nano size facilitates the penetration of different live tissues, into the body, through the blood circulatory system [21]; the NPs can be distributed in various organs thus accumulating as foreign bodies [22,23]. It has been shown that the TiO_2_-NPs can pass the testicular blood barrier inducing an effect on the testis and then on male reproductive health, given their nano-size [24,25,26]. Several studies in rodents and mice have shown the toxicity effect of TiO_2_-NPs on the male reproductive system; in addition, there is an increase in data about in vitro and in vivo studies on NPs that support the notion that different types of nanoparticles are capable of altering the normal and physiological activity of the endocrine system [27]. According to European Food Safety Authority EFSA (2010), any substance that has the ability to interact with one or more elements of the endocrine system (i.e., exhibit endocrine activity) falls into the category of “endocrine active substances” also called “endocrine disrupting chemicals” (EDCs). Endocrine disruptors have the potential to dysregulate hormone activity in exposed aquatic organisms [28] and their disruption is linked to reproduction and development dysregulation [29]. Although several chemical products are recognized as potential endocrine disruptors, the research has been limited to a few groups of chemical substances (disinfection byproducts, perfluoroalkyl and polyfluoroalkyl substances, bisphenol A, phthalates, pesticides, pharmaceutical agents, and heavy metals); consequently, the data on a number of other xenobiotics that may act as EDCs are still scant and incomplete [30]. 

In this regard, ENPs must be included among these chemical compounds since the intensity of ENP exposure is significantly increased due to their applications; moreover, they are persistent in the environment. The release of ENPs into the aquatic environment along with other environmental contaminants is common [31].

Thus, in this study, we investigated the possible toxic effect of commercial titanium dioxide nanoparticles (TiO_2_-NPs) on embryonic development and the male reproductive system, evaluating markers that act in their possible role as endocrine disruptors.

## 2. Materials and Methods

### 2.1. Preparation of Work Solutions

The titanium dioxide nanoparticle powders supplied by CNR-IMM (Microelectronics and Microsystems of Catania-National Research Council, Italy), were purchased from Sigma Aldrich. (St. Louis, MO, USA) 

According to the data in the literature and the suggestions by researchers of the National Research Council, we chose the following concentrations to test: 1 mg/L, 2 mg/L, and 4 mg/L of TiO_2_-NPs [32]. Briefly, each mass (1 mg, 2 mg, 4 mg) was dispersed in 1 L of osmosis water which was reconstituted with the addition of inorganic salts (sea salt and red sea salt); this is optimal for the housing of zebrafish, according to Nüsselin-Volhard and Dahm [33] and prevents fungi growth. Four cycles of sonication were performed for each solution; each cycle duration was 20 min with a 10 min break using an ultrasonic bath (FALC Labsonic LBS2, Treviglio, BG, Italy) (with a frequency of 40 kHz under an extractor fan to disrupt any possible aggregates [34].

### 2.2. Breeding of Zebrafish and Experiment Design

*Danio rerio* fishes were raised in a fish room at the Fish Pathology and Experimental Centre of Sicily (CISS) of the Department of Veterinary Science (University of Messina). The embryos were used to perform the zebrafish embryo toxicity test (ZFET); whereas the male adults had been used for a 30-day chronic toxicity test (authorization n°1244/2015-PR approved by the Italian Health Ministry).

Male and female adults were useful for the supply of embryos and were kept in a breeding room in an optimal condition regarding photoperiod (light/dark cycle:14 h/10 h), water quality (27 ± 1 °C, pH 7.2 ± 0.3, 6.00 ppm dissolved oxygen content (DO)), and feeding. Subsequently, male and female fishes (ratio 2:1) were placed in a hatching tank to breed. The tank was equipped with steel grids for the eggs to fall through to the bottom of the tank and avoid predation by the adults. The eggs were collected by Pasteur pipettes, rinsed in aquarium water at 28 °C, and analyzed under a stereomicroscope (≥ 30-fold magnification). The infertile eggs were discarded, and the fertilized eggs at the blastula stage (about 3–3.5 h post-fertilization) were selected to perform the zebrafish embryo toxicity test (ZFET).

One- to two-year-old zebrafish wildtypes, not consanguineous, with a body weight of about 0.5 g and an average length of 3 cm, were used for a 30-day chronic toxicity test. Prior to TiO_2_-NP treatment, fishes were acclimated to experimental conditions (26–28 °C; light: dark/14 h:10 h; daily water change), including daily manipulation and nutrition. After, the zebrafish were randomly divided into four groups of 10 fishes: three experimental groups (1 mg/L, 2 mg/L, and 4 mg/L TiO_2_-NPs) and a control group (with osmosis water).

### 2.3. Acute Toxicity Experiment of Zebrafish Embryo

The assay was conducted in accordance with the guidelines of the fish embryo acute toxicity (FET) test with the zebrafish (*Danio rerio*) developed by the Organization for Economic Cooperation and Development (OECD TG 236) [35,36,37]. As suggested by protocol procedure, 24 eggs at the blastula stage were transferred into a 24-well multi-plate with one embryo per well, containing 2 mL of work solutions at 28 °C. Multi-well plates were set up for the 1 mg/L, 2 mg/L, and 4 mg/L TiO_2_-NPs solutions, in addition, multi-well plates of positive controls (3,4-dichloroaniline at the concentration of 4 mg/L in water) and negative controls (water dilution) were made. Three replicates were performed for each experimental group. The maintenance of 26 ± 1 °C in wells was ensured by a control of room temperature; moreover, every 24 h each work solution was renewed in all wells (semi-static renewal) [35]. The exposure time was selected to be 96 h pos-fertilization (hpf) because even if most organs in the embryos are well developed at 96 hpf, the larvae are formed after 120 hpf [38,39]. We selected the end of the test to be at 144 h after fertilization to investigate the expression of protein markers associated with the endocrine disruption by immunohistochemical analysis.

### 2.4. Evaluation of Toxicological Endpoints and DanioScope™ Analysis

According to the OECD, every 24 h the acute toxicological endpoints (coagulated embryos, lack of somite formation, non-detachment of the tail, and lack of heartbeat) were assessed and quantified as observed or not observed; any positive outcome in one of these observations would indicate that the zebrafish embryo was dead. All endpoints can occur after 24hrs of exposure, except for heartbeat which in normal zebrafish development is visible after 48 h. In addition, using DanioScope™ software (Noldus Information Technology bv, Wageningen, the Netherlands), the following were evaluated: heartbeat, body length of larvae, and malformations (Supplementary Information Text S1).

### 2.5. Immunohistochemical Analysis on Zebrafish Larvae

An immunohistochemical analysis was performed to localize (in whole larvae) a marker of oxidative stress heat shock protein-70 (Hsp70), poly (ADP-ribose) polymerase-1 (PARP-1), and a marker that suggested the action of TiO_2_-NPs such as endocrine disruptor sex hormone-binding globulin (SHBG) and prothymosin-α (PTMA). Moreover, metallothionein, a specific marker of exposure to TiO_2_-NPs, was detected. The procedure was based on the immunofluorescence protocols of Pecoraro et Colleagues [37] (Supplementary Information Text S2).

### 2.6. Adult Zebrafish Exposure Experiment

As previously mentioned, adult zebrafish were randomly divided into four groups of 10 fishes: three experimental groups (1 mg/L, 2 mg/L, and 4 mg/L TiO_2_-NPs) and a control group (with TiO_2_-NPs osmosis water). The tanks were equipped with aerators and fishes were subjected to a semi-static exposure regime for 30 days (water was changed every 24 h with a new solution of TiO_2_-NPs). The water parameters were monitored daily before and after the replacement of the solutions by the multiparameter probe (HI9829 Aquaprobe, Hanna instruments, Padua, Italy). During experimentation, the fishes were maintained as follows: photoperiod 10 h dark/14 h light with an intensity equal to 250 lux, 6.9–7.5 pH, 26–28 °C temperature, and dissolved oxygen ≥6.00 ppm; in addition, they were fed with commercial feed “GEMMA micro 300 Skretting Zebrafish” twice per day prior to the change of water TiO_2_-NPs solution to avoid adsorption of nanoparticles by food particles. After adding the food, fishes were monitored for 10 min to verify that the food had been consumed. After the change of TiO_2_-NPs solution, the fishes’ behavior was evaluated for one-hour to highlight changes in swimming speed, respiration, loss of equilibrium, bottom stationing, and any other possible abnormal behavior. Until the end of the experimentation (30 days), the zebrafish were kept under the conditions mentioned and without sources of noise and/or vibrations. Upon completion of the experimentation, the fishes were euthanized by anesthesia with a dose of 0.7 g/L tricaine methane sulfonate (MS-222), buffered, then testis and gill tissues were dissected.

### 2.7. TiO_2_-NPs Accumulation

Through the single particle inductively coupled plasma-mass spectrometer (spICP-MS), the concentration of TiO_2_-NPs in organs of zebrafish was evaluated. TiO_2_-NPs were analyzed using ICP-MS NexION^®^ 350D (Perkin Elmer, Waltham, MA, USA) with the Syngistix Nano Application software (Perkin Elmer, Waltham, MA, USA). Supplementary Information Text S3.

### 2.8. Histological Examination

Histological examination was performed following our standard protocol (Supplementary Information Text S4). The sections were observed using an optical microscope (Set E200 Nikon, Amsterdam, Netherlands)) and the images were captured by a digital camera (CMOS Nikon, Amsterdam, Netherlands) connected to the microscope. Potential morphological alterations on the structure of the testis, and gill tissues were identified.

### 2.9. Immunohistochemical Analysis

An immunohistochemical analysis was performed on testis sections to detect SHBG and P540 expression. This followed our standard protocol of immunohistochemical used for several experiments on zebrafish [32]; furthermore, the same primary antibodies used for the immunohistochemical investigation on zebrafish larvae were used.

### 2.10. Preparation of Semithin Sections and Electron Microscopy

Tests were fixed with 2.5% glutaraldehyde (brand) for 90 min at +4 °C. Subsequently, the protocol for electron microscopy was followed (Supplementary Information Text S5) to obtain semi-thin (thickness 0.85 µm) and ultra-thin (thickness 0.085 µm) sections useful for transmission electron microscopy (TEM,). (Palackého třída, Brno, Czech Republic),

### 2.11. RNA Extraction and qRT-PCR 

The qRT-PCR was used to evaluate the mRNA levels of marker genes in the testis. SHBG, SRD5A2, SOD2, and GPX4B were selected for analysis, and β-actin was used as a housekeeping gene (Supplementary Information Text S6).

### 2.12. Crystal Structure of Sex Hormone-Binding Globulin (SHBG)

The SHBG protein in zebrafish, is ortholog to human. Thanks to Fingerprint for Ligand and Protein FLAP, it was analyzed that SHBG bound to dihydrotestosterone (DHT) (Supplementary Information Text S7).

### 2.13. Statistical Analysis

Data on zebrafish embryo experimentation, namely the coagulate, survival, and hatching rate of the TiO_2_-NP exposed groups and the unexposed group, were represented as the average percentage of the coagulate, survival, and hatching rate from three replicates. Statistical analysis was performed by one-way analysis of variance (ANOVA) test to compare differences between groups. A *p* < 0.05 was considered to be a statistically significant difference.

## 3. Results

### 3.1. Nanoparticles Characterization

**Figure 1:**
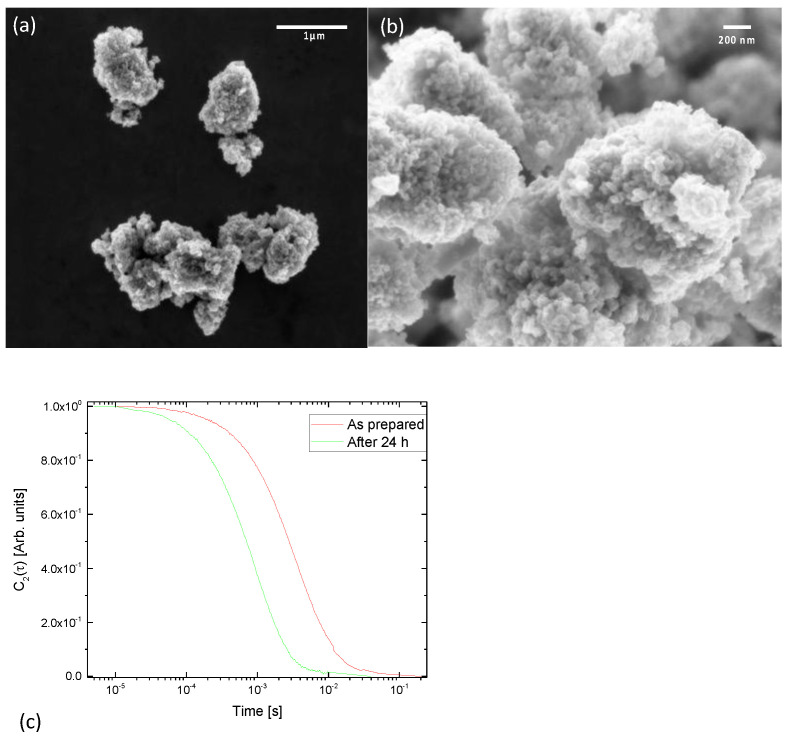
SEM images of TiO_2_-NP. Marker is 1 µm (**a**), and 200 nm (**b**). (**c**) Autocorrelation function of the colloidal solution as prepared solution and after 24 h of sedimentation.

According to the manufacturer’s information, the purity of the TiO_2_-NPs was 99.5% with metal traces. The nanopowder crystalline phase was mixed: 86% anatase, and 14% rutile. SEM analyses were performed in plan-view to characterize the morphology of the nanoparticles (Figure 1). As is apparent from the SEM analyses, powders are constituted by ‘cobblestones’ with a size in the order of 1–2 microns (Figure 1, at left); nevertheless, at higher magnification, SEM images show that these structures are composed by smaller particles aggregated to each other. Isolated nanoparticles had an average diameter of about 50 nm.

To characterize the nanoparticle solution, hydrodynamic diameter was measured by dynamic light scattering. Apparatus and methods are described elsewhere [40,41].

Figure 1c shows the dynamic light scattering DLS autocorrelation function for the “as prepared” solution and for the solution after 24 h of sedimentation. The hydrodynamic diameter was estimated to be 1100 nm for the “as prepared” solution (red curve). This is in perfect agreement with the SEM image of Figure 1a. Furthermore, solutions were allowed to sediment for 24 h and correlation functions were measured again. The hydrodynamic diameter decreased to about 283 nm (green curve). This indicates that the larger particles were deposited and only smaller aggregates remained in suspension during the experiment.

### 3.2. Embryonic Development of Zebrafish

The toxicity of TiO_2_-NPs on zebrafish embryos was defined by observing specific toxicological endpoints, as mentioned. At 24 hpf, the rate of coagulated eggs was 11.3% for the 1 mg/L group, 28.3% for the 2 mg/L group, and 23.3% for the 4 mg/L group, whereas, in the unexposed embryo group was 5%. At 48 hpf, the coagulated egg rate increased for the 4 mg/L groups (25%); otherwise, it remained unchanged in the other experimental groups (see Supplementary Informations Figure S1).

**Figure 2:**
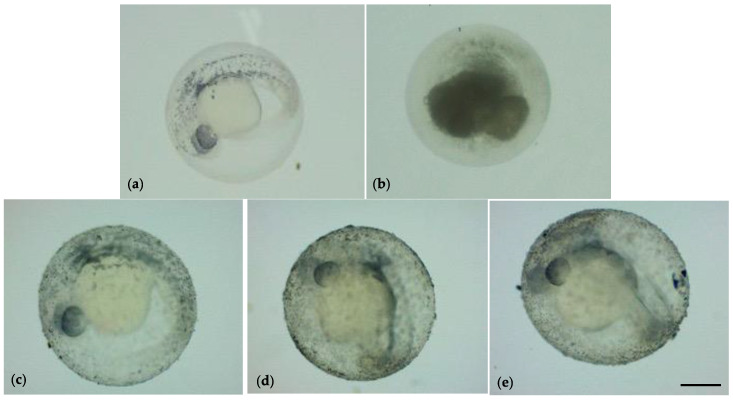
(**a**) Zebrafish embryo unexposed to TiO_2_-NPs (negative controls); (**b**) zebrafish embryo exposed to 3,4-dichloroaniline (positive controls). Zebrafish embryo exposed to 1 mg/L (**c**); 2mg/L (**d**); and 4 mg/L (**e**) TiO_2_- NPs. Scale bar 410 µm.

At 24 hpf, and all the exposure period, TiO_2_-NPs were evidently deposited on the bottom of the wells. However, TiO_2_-NPs also adhered on the surface of embryonic chorion with increasing concentrations of TiO_2_-NPs, as shown in Figure 2

**Figure 3:**
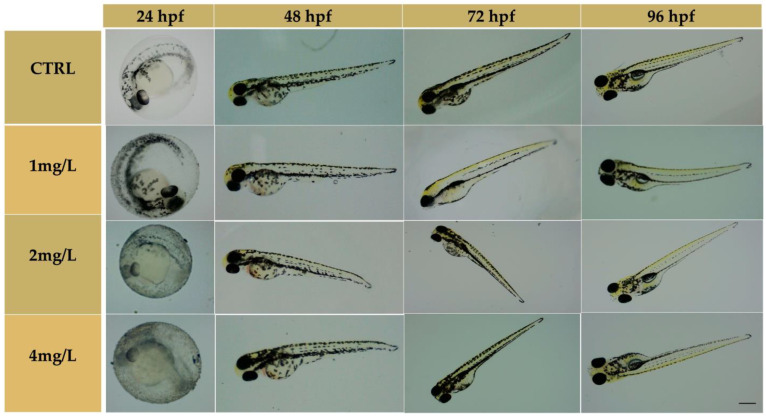
Phenotypes of larvae exposed to TiO_2_-NPs and the unexposed group from 24 to 96 hpf. Scale bar 420 µm.

The TiO_2_-NPs formed an external white layer on the chorion, that did not affect the hatching of embryos. At 48 hpf, the hatching rate was 36.7% (1 mg/L), 15% (2 mg/L), and 18.3% (4 mg/L); whereas the unexposed group showed a rate of 12%. The hatching rate was statistically significant between all exposed groups and the unexposed group (*p* < 0.05); no statistical significance had been observed at 72 hpf except for the 1 mg/L group (see Supplementary Information Figure S2). After 96 h of exposure to TiO_2_-NPs, the survival of the hatched embryos for control was above 90%, as described by Kimmel et al. [38], while the survival of the exposed groups was below 90% but remained unchanged until the end of experimentation. No statistically significant increase in mortality rates for groups exposed to 2 mg/L and 4 mg/L TiO_2_-NPs relative to the control treatments were observed (*p* > 0.05). At the end of the test (144 hpf), we observed a rate of mortality of 8.30% (unexposed group), 13.30% (1 mg/L), 31.7% (2 mg/L), and 25% (4 mg/L). All embryos showed a complete development of head, notochord, fin, pigmentation, and the organ’s heart and eyes. There was no morphological malformation compared to the unexposed group (Figure 3). 

### 3.3. DanioScope Analysis

Through the DanioScope software (1.2 Wageningen, the Netherlands)), it was shown that TiO_2_-NPs affected the body length of larvae because they exhibited reduced body length compared to the unexposed group (*p* < 0.05) at 96 hpf. The mean body length in the 4 mg/L group was 172 µm, while in the unexposed group was 215 µm. The heart rate was measured through the registration of beats per minute (BPM) by the DanioScope software which highlighted an increase in embryos exposed. An increase in BPM was observed in the 1 mg/L group (217.20 BPM) and a higher BPM (236.3) appeared in the 4 mg/L group. BPM in zebrafish is physiologically around 120–180 bpm. The exposure to TiO2-NPs resulted in a statistically significant increase in heart rates in exposed embryos (*p* < 0.05) (see Supplementary Figure S3).

### 3.4. Immunohistochemical Markers on Zebrafish Larvae

**Figure 4:**
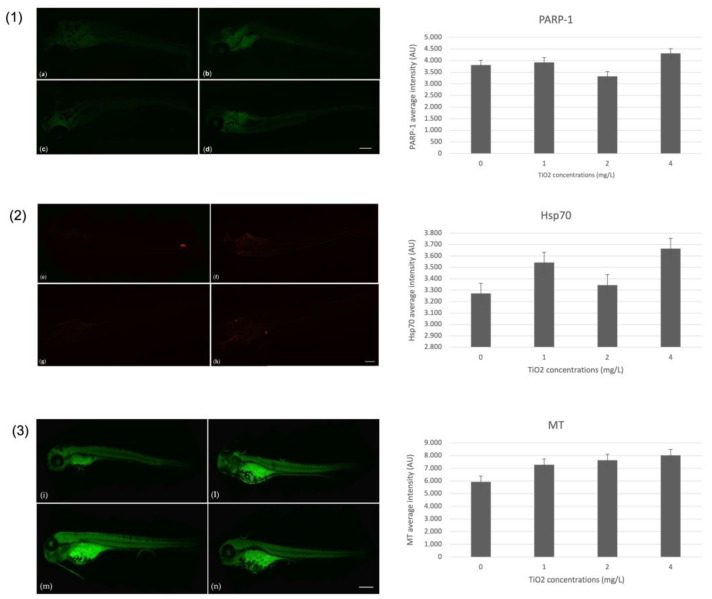
In order (1) PARP-1 antibody-staining, (2) Hsp70 antibody-staining, and (3) MT antibody-staining. (**a**,**e**,**i**) larva unexposed to TiO_2_-NPs; (**b**,**f**,**l**) larva exposed to 1 mg/L TiO_2_-NPs; (**c**,**g**,**m**) larva exposed to 2 mg/L TiO_2_-NPs; and (**d**,**h**,**n**) larva exposed to 4 mg/L TiO_2_-NPs. The histogram next to each photo represents the average fluorescence intensity (AU) of the corresponding biomarker. Scale bar 420 µm.

For markers of oxidative stress, the immunohistochemical investigation showed a positivity for the poly (ADP-ribose) polymerase-1 (PARP1). Positivity was observed at the lower concentration (1 mg/L) and increased at the higher concentration (4 mg/L) compared to the control. Moreover, a positivity for heat shock proteins-70 (HSP70) in the exposed group, especially at the higher concentration, suggests the ability of TiO_2_-NPs to induce oxidative stress. Additionally, positivity for biomarker metallothioneins (MTs), which is linked to detoxification pathways in the presence of toxic substances, was found in the whole body of the embryo except for the end of the tail. Using Image J software, the intensity of fluorescence was quantified for each biomarker evaluated. Figure 4 shows the images of larvae and their average fluorescence intensity (AU). 

**Figure 5:**
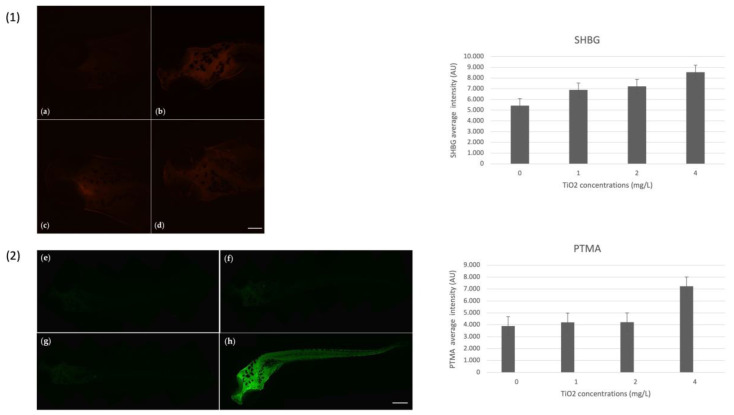
In order (1) SHBG antibody-staining and (2) PTMA antibody-staining. (**a,e**) larva unexposed to TiO_2_-NPs; (**b,f**) larva exposed to 1 mg/L TiO_2_-NPs; (**c,g**) larva exposed to 2 mg/L TiO_2_-NPs; and (**d,h**) larva exposed to 4 mg/L TiO_2_-NPs. The histogram next to each photo represents the average fluorescence intensity (AU) of the corresponding biomarker. Scale bar 420 µm.

Regarding the SHBG and PTMA, positivity was observed for both. Particularly for SHBG, positivity was observed on the head of the embryo, with a higher expression for the 4 mg/L TiO_2_-NPs concentration compared to the control. Whereas, the positivity for prothymosin α (PTMA) occurred in the body of larvae, especially at the concentration of 4 mg/L. Figure 5 shows the images of larvae and their average fluorescence intensity (AU).

### 3.5. Adult Exposure

Daily monitoring until the end of experimentation, revealed no fish died, no abnormal behaviors such as loss of equilibrium, refusal to feed, and no apparent abnormalities in the body of fish. An accumulation of TiO_2_-NPs was found in zebrafish testicles of our experiment groups at concentrations of 1.18 × 10^−3^ mg/Kg and 8.14 × 10^−3^ mg/Kg under 2 mg/L and 4 mg/L doses, respectively. Using the Syngistix Nano Application software, it appeared that TiO_2_-NPs had a size < 100 nm. In our study, 30-day exposure of zebrafish to TiO_2_-NPs suggests that fish uptake TiO_2_-NPs by breathing and feeding. The nanoparticles were constantly resuspended in the aqueous medium, so fish are highly likely to internalize them via their gills and mouth. We found an accumulation of TiO_2_NPs in the gill tissue for the concentrations of 2 mg/L (1.50 × 10^−3^ mg/Kg) and 4 mg/L (2.39 × 10^−3^ mg/Kg); whereas, no accumulate TiO_2_-NPs were found in the control as expected, but also at 1 mg/L concentration.

### 3.6. Histological Observations

**Figure 6:**
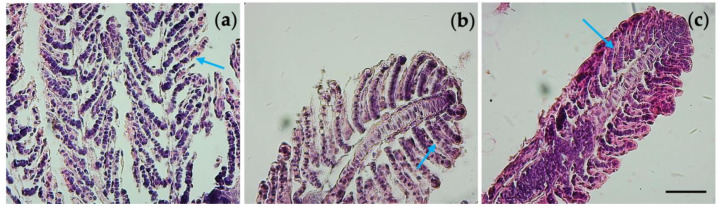
Histological section of gills. (**a**) CTRL gills. Exposed groups to (**b**) 2mg/L; and (**c**) 4mg/L TiO_2_-NPs. Arrows indicate the secondary lamellae, that showed a hyperplasia in the exposed groups, while it is not appeared in the control groups. Scale bar 1500 µm.

Gill tissue from the control, 2 mg/L, and 4 mg/L groups showed alterations in their morphology. We observed increasing cellularity in the interlamellar space and hyperplasia brings a higher width of the secondary lamella with respect to control (indicated by blue arrows in Figure 6.

**Figure 7:**
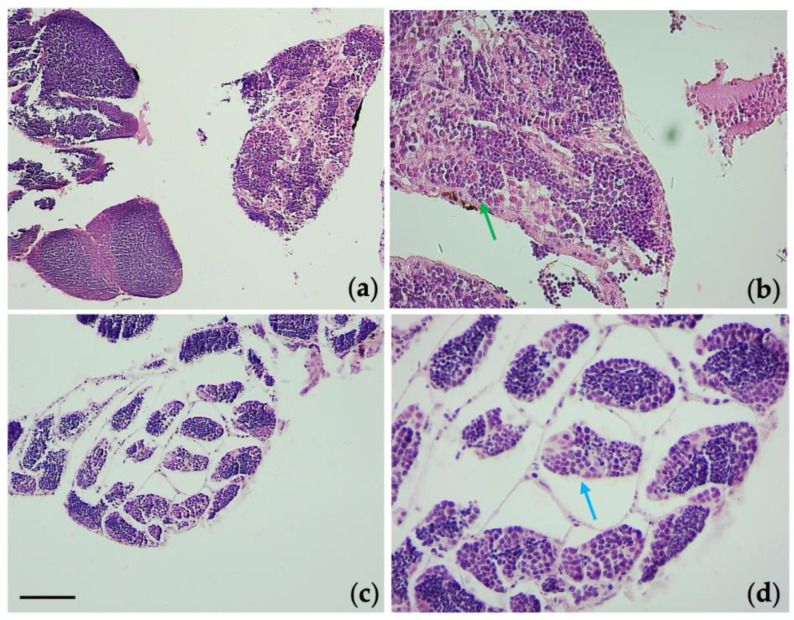
Histological sections of the testis. Unexposed group: 10× (**a**), and 40× (**b**). Group of 2mg/L TiO_2_-NPs: (**c**) 10×, and (**d**) 40×. E-E staining, sections 4 µm. Blue arrow indicate the detachment of the spermatogenic epithelium from the connective tissue in the exposed group (2mg/L), while in the control group the spermatogenic epithelium maintains its contact with connective tissue (green arrow). Scale bar 260 µm.

**Figure 8:**
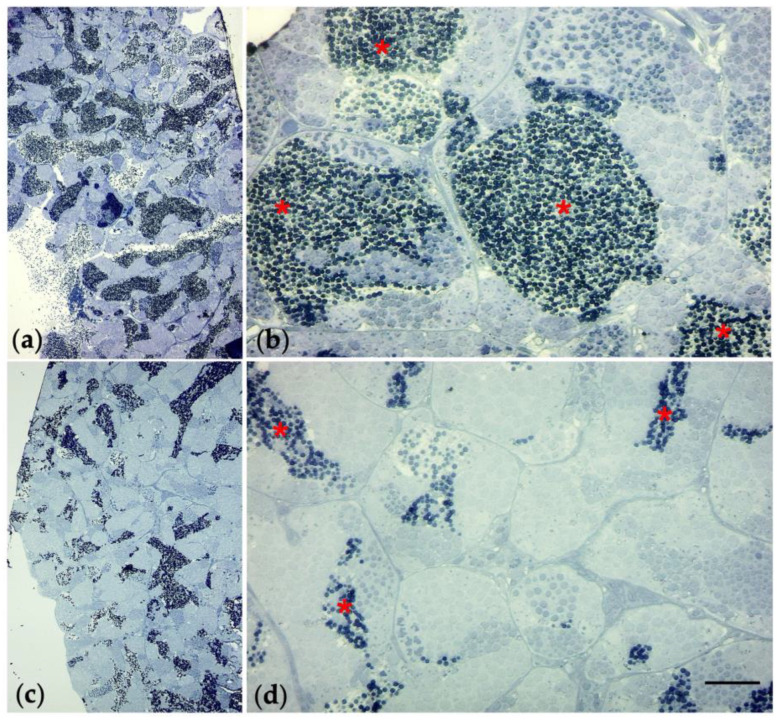
Histological sections of testis staining with toluidine blue (sections 0.85 µm). Unexposed group: 10× (**a**), and 40× (**b**). Group of 2mg/L TiO_2_-NPs: (**c**) 10×, and (**d**) 40×. Red * indicates the area occupied by spermatozoa into the tubules. Scale bar 260 µm.

Examination of the gonads tissue showed an alteration in the spermatogenic epithelium in the groups exposed to TiO_2_-NPs. The exposure of TiO_2_-NPs caused a detachment of the spermatogenic epithelium from the connective tissue, whereas, it had not been observed in the control group, respectively, blue arrow and green arrow in Figure 7. Moreover, the tubules (the area occupied by spermatogonia) were increased, compared to the area occupied by spermatozoa that were decreased (in Figure 8, red * indicates the area of spermatozoa, which have blue nuclei; whereas, the remaining are spermatogonia, which have light blue nuclei).

**Figure 9:**
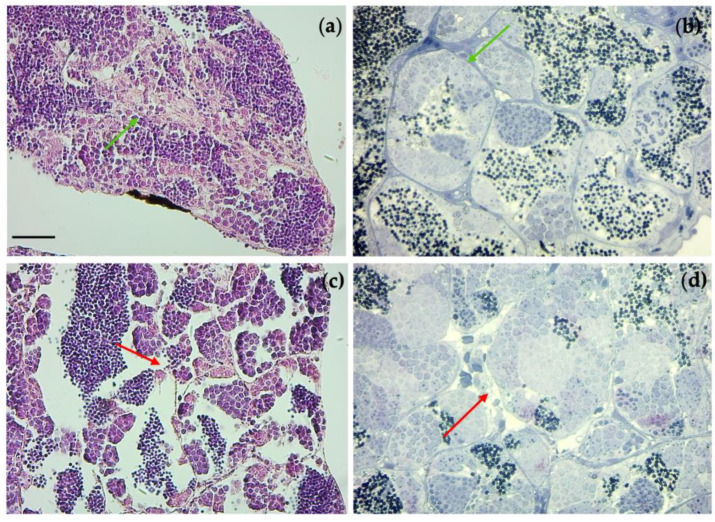
(**a**) (10×) and (**b**) (40×) unexposed group with good morphology and organization of tubules testis; (**c**) (10×) and (**d**) (40×) group exposed to 4mg/L TiO_2_-NPs with disorganization of tubules testis. Green arrows indicate the intact and well organization of tubules testis, while the red arrows indicate their disordered organization. Scale bar 260 µm.

A disordered arrangement of spermatogonia was observed at the concentration of 4 mg/L (red arrow), and the connective tissue presented irregularities making it difficult to distinguish between Leydig cells and connective cells. Otherwise, the unexposed group was intact (green arrow) (Figure 9). Whereas, the testis tissues in the 1mg/L dosage group showed no changes compared with that of the control.

**Figure 10:**
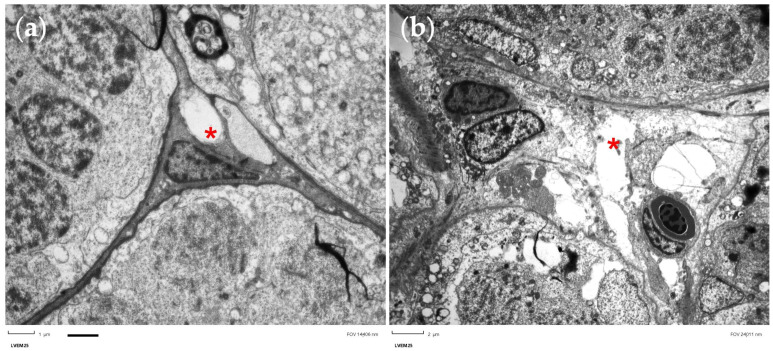
Ultrathin sections of zebrafish testis. (**a**) Section of testis unexposed; (**b**) section of testis exposed to 4 mg/L with evident vesiculation. Red * indicate the Sertoli cells that showed a evident vesiculation in the exposed group compared to control. Scale bar 2 µm.

In the ultrathin sections of testis, we observed by transmission electron microscopy (TEM) the presence of vesiculation in Sertoli cells. It was evident at the concentration of 4 mg/L with the detachment of the cell membrane compared to the control, as shown by red * in Figure 10.

### 3.7. Immunohistochemical Analysis and Gene Expression

**Figure 11:**
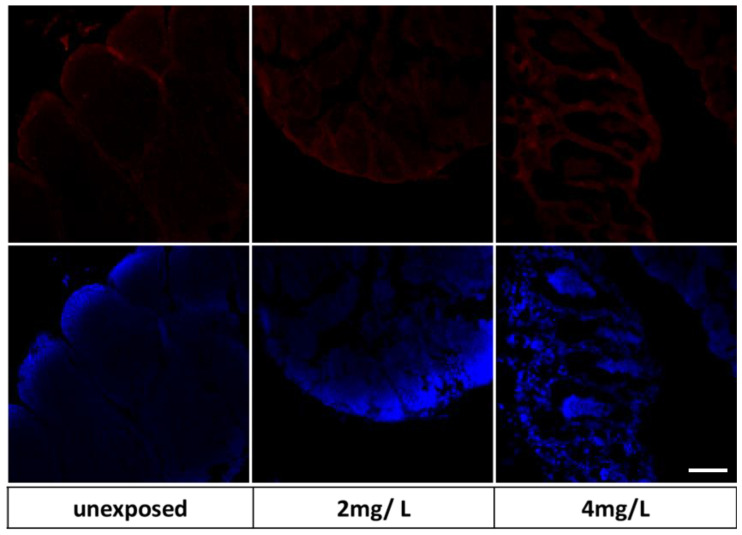
SHBG expression in testis tissue. Nuclei blue (DAPI) and red fluorescent of SHBG protein. Scale bar 265 µm.

We explored markers to investigate the action of TiO_2_-NPs such as endocrine disruptors. In particular, the results of immunohistochemical analysis on SHBG reveal that it was expressed in control groups in a regular way because the cysts maintained their morphological organization; however, positive expression remained in the group exposed to 2 mg/L, than in 4 mg/L. As shown in Figure 11, positivity was related to the cells that were around the seminiferous tubule cysts.

**Figure 12:**
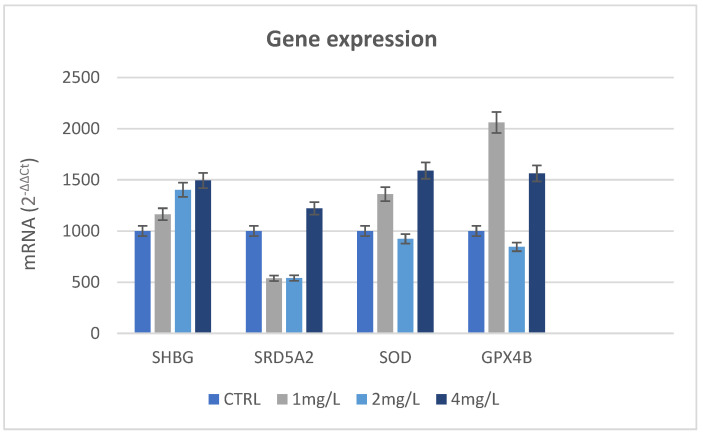
Results of qRT-PCR of all genes investigated.

The result of gene expression of SHB in the testis indicates an increased expression of the SHBG gene for the 2 mg/L and 4 mg/L concentrations compared to the control group. Increased transcription of antioxidant enzymes such as glutathione peroxidase (GPX), and especially superoxide dismutase (SOD), was observed. Figure 12 shows the results of qRT-PCR of all genes investigated. 

In this way, oxidative stress is a common pathway of toxicity and disease that may be caused by many pollutants, such as TiO_2_-NPs. 

**Figure 13:**
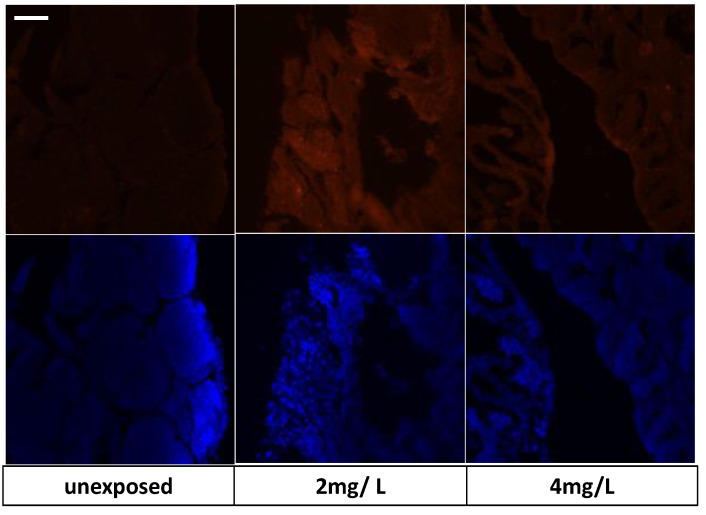
P540 expression in testis tissue. Nuclei blue (DAPI) and red fluorescent of P540 protein. Scale bar 265 µm.

The immunohistochemical investigation of cytochrome P540 (Cyp19b) confirmed the ability of nanoparticles to induce oxidative stress. As shown in Figure 13, positivity was found in the expression of P540 in the groups exposed to TiO_2_-NPs (2 mg/L and 4 mg/L concentrations) compared to the control which did not show positivity for the biomarker.

### 3.8. Crystal Structure of Sex Hormone-Binding Globulin (SHBG)

**Figure 14:**
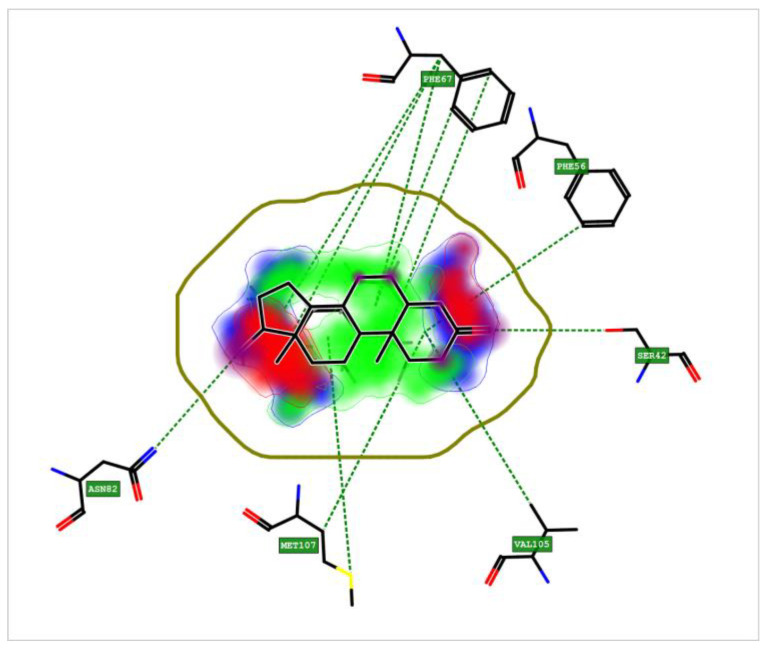
2D-depiction shows the interactions that characterize the pocket.

The result of the crystal structure of the SHBG shows that the pocket is very large and many amino acid residues are involved. Figure 14 shows the SHBG interactions involved in binding, which can characterize the pocket. The interaction most involved is that of the hydrophobic character (green area), and there are also two areas of hydrogen bond acceptor characters (red). Thus, the hydrophobic interaction could explain the binding to NPs.

## 4. Discussion

In this study, the toxicity of TiO_2_ NPs on zebrafish embryos and adult fish has been investigated. With the zebrafish embryonic test (Z-FET), it was made evident that TiO_2_-NPs did not interfere with embryonic development because zebrafish chorion acted as a special biological structure that covered the embryo until hatching. Then, it acted as a barrier that blocked the entry of various pollutants [42]; simultaneously due to its pores, the chorion ensured the transport of necessary oxygen, salt ions, and nutrients from the aquatic environment to the embryo and excretion of waste in the opposite direction [43]. The small diameter of pores (between 300 nm and 1 micron) can allow the entry of NPs adhering to the chorion [43,44]. NPs diffusion may be toxic to embryo development during the period of organogenesis [45]; however, the literature is scant regarding the interaction of NPs with the chorion, and how this structure interacts and affects the absorption, accumulation, and distribution of nanoparticles in the embryos [46].

Although it was evident that there was sedimentation of TiO_2_-NPs during the experimentation, the embryos and larvae were constantly exposed to the TiO_2_-NP aggregates because they were mostly located on the bottom of the wells, also after the hatching when they could freely swim. TiO_2_-NPs have low acute toxicity to fish survival [47] and TiO_2_-NPs concentrations higher than our experimental groups did not affect the survival rate in zebrafish [48]. In addition, the group control showed normal development [38] as well as the exposed groups with a low dose (1 mg/L) of TiO_2_-NPs [49]. However, using the DanioScope software, it was shown that TiO_2_-NPs caused alterations in the body length of larvae and the heart rate. These data are in accord with other studies on nanoparticle toxicity [50,51,52,53,54]. Regarding immunohistochemical markers on zebrafish larvae, the positivity of the biomarkers whose expression is regulated by environmental stressors highlights that the TiO_2_-NPs are stressful stimuli for zebrafish embryos. Despite this, the zebrafish larvae are able to resist the presence of toxic substances and they can tolerate the presence of metal concentrations. In particular, the PARP-1 is involved in single-strand break (SSB) repair [55] induced by chemicals [56,57,58], the heat shock protein-70 (Hsp70) expression increases in response to environmental and physiological stressors [59] and protects the cells against induction of cell death by a variety of stresses; data in the literature have shown an increased expression of the Hsp70 due to exposure of chitosan nanoparticles, ZnO [60], and transition metals oxide (CuO, ZnO, NiO, and Co3O4) [61]. Finally, metallothioneins (MTs) are involved in homeostasis, protection against heavy metals and oxidant damages, and metabolic regulation, sequestration, and/or redox control [62]. Previous studies have shown the positivity of MTs in zebrafish embryos exposed to AuNPs [63]. 

Positivity for the SHBG and PTMA biomarkers can suggest that TiO_2_-NPs act similar to endocrine disruption. The SHBG is a protein capable of binding steroids in the blood of fish and other vertebrate species. It is well characterized in humans [64,65] but only one ortholog (SHBG) has thus far been identified in zebrafish; being expressed in the digestive tract, liver, gills, pancreas, and testis [66,67]. Furthermore, on sex steroid transportation, regulation, and action [68], the SHBG has shown affinity to synthetic steroids such as ethinylestradiolor gestagens [66,68] and, in addition, it has been reported that it binds with phthalates and other environmental contaminants [69,70,71]. In this regard, SHBG is a potential target for environmental compounds found in the body. Consequently, increasing the risk of potential disruption in steroid homeostasis, experimental exposure to phthalate in rodents has shown adverse effects due to the deregulation of metabolic pathways by phthalate compounds [72]. Regarding fish, there is scant knowledge about SHBG structure and the site(s) of expression, although studies have highlighted that during the development of zebrafish, SHBG mRNA first appears within the liver and gut [68]. In addition, studies have shown SHBG affinity to xenobiotics able to act as endogenous sex steroids (testosterone and estradiol). Chen et al. [73] have found an increase in SHBG in zebrafish larvae exposed to bisphenol AF (BPAF), which is recognized as an endocrine disruptor. Prothymosin α (PTMA) is a small nuclear protein (109–113 amino acids depending on the species) with a potent nuclear localization signal (NLS), although data show cytoplasmic and extracellular presence as well, under specific physiological or pathological conditions [74,75]. It was observed that PTMA expression levels vary following stimulation by estrogen; thus, as well as SHBG expression, the positivity increment of PTMA in embryo zebrafish in our experimentation suggests that TiO_2_-NPs act similarly to endocrine disruption. 

In adult exposure, the accumulation of TiO_2_-NPs in the testis can lead to impairment of the male reproductive system, as suggested by data in the literature. The accumulation of TiO_2_-NPs in the testis induces cytotoxicity and gene expression changes [76,77,78,79,80,81,82]. The exposure to the TiO_2_-NPs can occur via various routes; generally, the NPs enter and distribute in the exposed site, but due to the blood they are translocated to secondary organs such as the liver, spleen, kidneys, brain, ovaries, and testes [83,84,85,86]. As previously mentioned, it is highly likely that the fishes internalize the TiO_2_-NPs via their gills and mouth. Gills are usually targeted organs for toxicity because they are in continuous contact with the water column; therefore, they are the main entrance route for all contaminants and for nanomaterials [83]. Into the intestine, bioaccumulation of TiO_2_-NPs was lower than in gills. Since TiO_2_-NPs after ingestion through the mouth were distributed in the digestive tract and then excreted through feces [85], there was no oral supply of nanoparticles because no oral administration was performed.

The gills’ histological alterations that were observed are in accordance with the literature; the exposure to copper nanoparticles had reported a 3.5-fold increase in the gill filament of zebrafish, at already 24 h of exposure [86]; whereas, silver nanoparticles caused a slight change. In addition, the NPs are foreign substances taken by mononuclear phagocytic cells which become the entry route of NPs into the tissues and cells. The tissue macrophages phagocytose and sequester nanoparticles; for example, in the mouse model, it was demonstrated that there was a durability of Au, Ag, and SiO_2_ NPs thanks to their action. This probably occurred in our experiment because, as previously mentioned, bioaccumulation of TiO_2_-NPs was found in the testes of our experimental groups. Our results have shown an alteration in the spermatogenic epithelium in exposed groups to TiO_2_-NPs. Previous studies on mice treated intragastrically with dosages of 10, 50, and 100 mg kg^−1^ of body weight (PC) anatase TiO_2_-NPs for 28 days showed morphological changes in testes with a reduction in germ cell number, spherospermia, interstitial glands vacuole, malalignment, and vacuolization of spermatogenic cells [87]. In zebrafish, negative effects on the epithelium of testicular tubules have been observed with parenchyma degeneration, a decline in germinal epithelium cells and spermatozoa, or reduction in spermatogonial differentiation, particularly in exposure to chemical substances [88,89,90]. Few previous studies have evaluated the negative effect of TiO_2_-NPs on zebrafish testis; however, Kotil et al. [32] evaluated the ultrastructure of zebrafish testis exposed to 1 mg/L, 2 mg/L, and 4 mg/L concentrations of TiO_2_-NPs. Their results showed that TiO_2_-NPs induced autophagy and necrosis at higher doses in Sertoli cells and consequently negatively affected spermatogenic cells and testicular morphology. Our results are in accordance with the literature.

Regarding the positivity of the SHBG biomarker in the seminiferous tubule cysts, it is known that SHBG is in the blood of all vertebrate species, apart from birds, and acts as a carrier of androgens and estrogens regulating their bioavailability [91]. In mammalians, the testis produces an SHBG homolog known as the testicular androgen-binding protein (ABP) which is produced and secreted by Sertoli cells primarily under the influence of follicle-stimulating hormone and then secreted into the seminiferous tubular lumen to regulate androgen availability in the male reproductive tract [92]. Studies of the SHBG steroid-binding characteristics in several fish species [93,94] have shown that its affinity for endogenous sex steroids (testosterone and estradiol), and xenobiotics [95,96,97] varies between species; moreover, some evidence showed that plasma SHBG levels fluctuate in fish during the reproductive cycle [98,99]. In zebrafish, the tissue distribution of SHBG transcript was detected in the digestive tract and hepatopancreas; in addition, a low expression was detected in the testis using RT-PCR [68]. However, several data indicate, in agreement with the literature about mammalians, that the liver is the main expression site of SHBG in teleost fish [100,101]. In some species, it was also found that there was a significant expression of SHBG in several other tissues, which suggests that the circulating SHBG could have an extra-hepatic origin. In mammals, a local expression of SHBG in several target organs has also been evidenced and associated with a modulation of the steroidogenic signal [102]. Similarly, non-hepatic expression sites of SHBG in teleost could also be associated with local action in target organs. For this reason, the transcription of SHBG in fish could depend on different variants which have to still be identified. It is not known how the expression of the SHBG gene is regulated [68]. Our results evidence that an expression of SHBG could be increased in the presence of NPs. This was supported by the result of gene expression. Unfortunately, the biological importance of SHBG in fish is not studied as well as it is in mammals. It may be reasonably hypothesized that the function of SHBG proteins expressed locally in target organs and tissues could be different from the circulation of SHBG. As in mammals, fish SHBG protein is involved in sex steroid transport, regulation, and action [103]. Considering our evidence, SHBG could improve the spermatogenesis process because their localization in the testis could bring a higher intake of androgen hormone or TiO_2_-NPs could act similarly to androgen hormones. It could be supported by the result of the crystal structure of the SHBG, as shown in Figure 14, additional in support of the action of TiO_2_-NPs like androgen hormones are the result of gene expression on the SRD5A2 gene. This gene, in all vertebrates, encodes for the steroid enzyme 5-αreductase α-polypeptide 2 (SRD5α2), an enzyme of spermatogenesis [104], and it works by converting testosterone (T) to dihydrotestosterone (DHT). 5α-dihydrotestosterone (DHT) plays a physiologically important role in some fish species and it is associated with spermatogenesis. Several pieces of evidence have documented the consequences of a lack of SRD5α2 activity that brought a decrease of 5α-DHT levels in those tissues, but also negative effects on spermatogenesis and the structure of seminiferous tubules [105,106]. The lack of 5α-DHT mainly affects the Sertoli cells because they are involved in spermatogenesis by supporting germ cell development, as well as playing an important role in the structure of the seminiferous tubules and the maintenance of the blood-testis barrier. Nevertheless, our results differ from the negative effects due to the lack of enzyme activity. The expression of the SRD5α2 was increased in the gonads exposed to nanoparticles, and this consequently leads to its greater activity and production of DH. Then, exposure to TiO_2_-NPs does not alter spermatogenesis because they encourage the activity of enzyme SRD5α2. Similar data were observed in fish treated with 5α-reductase inhibitors (5ARIs) in which spermatogenesis is unaltered or even increased because levels of T and 11KT are increased [107,108]. Since the synthesis of SRD5αs is regulated by the androgens they produce, and considering our results, it can be hypothesized that nanoparticles may behave similarly to androgens. Unfortunately, no unified theory about how hormone levels change after exposure to NPs exists. Some studies showed that low-dose (1 mg/kg/dose) AgNPs intravenously injected into male CD1 mouse serum caused a significative increase of the intratesticular testosterone (T) [109]; whereas, it was discovered in another study that CeO_2_-NPs treatment caused decreases in T, FSH, LH, and prolactin (PRL) [110]. Indeed, these changes might be influenced by different factors such as particle type, size, and time of exposure. Therefore, research on how NPs affect hormones should be conducted and the lack of in vitro studies is still a problem. The cytochrome P540 (Cyp19b) confirmed the ability of nanoparticles to induce oxidative stress. CYPs are a large superfamily of enzymes capable of metabolizing several substances including steroids, pharmaceuticals, and xenobiotic compounds. They catalyze mixed-function oxidation reactions and the induction of their catalytic activity, measured as ethoxyresorufin-O-deethylase (EROD) activity, and expression (protein and transcript) is a useful biomarker of exposure to xenobiotics. Their catalyzing activity leads to the activation or inactivation of many endogenous and exogenous chemicals, with consequences for normal physiology and disease processes such as oxidative stress [111]. Nanomaterials of varying chemical compositions such as fullerenes, CNT, and metal oxides are able to induce oxidative stress [112]. Particularly in fish, TiO_2_-NPs cause oxidative stress with the production of reactive oxygen species (ROS) [85,112], and titanium dioxide nanoparticle aggregates (TiO_2_-NMs) cause oxidative stress in zebrafish embryos [48]. However, the investigations about oxidative stress by TiO_2_-NPs on testis are insufficient. Oxidative stress may be induced through oxidizing (e.g., hydrogen peroxide, H_2_O_2_) or photo-oxidizing (e.g., fluoranthene) agents that react with oxygen-producing reactive oxygen species (ROS) [113,114] Since the reactive oxygen species (ROS) are able to affect the physiology, growth, and survival in aquatic organisms [115,116], they, similar to mammals, developed an antioxidant defense system for neutralizing the toxic effects of ROS [117]. Antioxidant enzymes such as SOD, CAT, GPX, POD, and low-molecular-weight, nonenzymatic antioxidants (e.g., GSH) are important components of the antioxidant defense system in animals [118,119]. In our study, it is clear that TiO_2_-NPs are able to induce oxidative stress in the testis involving antioxidant defense [120,121,122,123]. The exposure to the lower concentration (1 mg/L) motivated the SOD to eliminate generated ROS as a protection mechanism against oxidative stress and a major increase was observed at the higher concentration (4 mg/L). Additionally, induction in the mRNA level of the GPX gene was higher at 1 mg/L concentration than at 4 mg/L. Fortunately, our results showed that the antioxidant defense of fish is induced by mild oxidative stress due to TiO_2_-NPs, and it does not overwhelm the detoxifying or antioxidant mechanisms.

## 5. Conclusions

In conclusion, through acute and long-term TiO_2_-NP exposure experiments on zebrafish, respectively, on embryos and adults, this research has improved knowledge of the action of TiO_2_-NPs on embryonic development and the male reproductive system.

We provide evidence that TiO_2_-NPs do not interfere with the development of zebrafish embryos, nor cause premature death in embryos; however, the embryos did show alterations in heartbeat, body length, and, above all, an increase in oxidative stress with the production of ROS and the expression of biomarkers associated to endocrine disruptors. The expression of the SHBG protein corresponding to the androgen binding protein (ABP) is increased in the presence of TiO_2_-NPs, both in zebrafish larvae and in male gonads. This result is important because ABP is known to increase with xenobiotics. An increase in gene expression has been recorded in the testes. Consequently, it can be assumed that the TiO_2_-NPs have an androgenic-like effect, as suggested by the increase in gene expression of SRD5A2, an enzyme that converts testosterone (T) to dihydrotestosterone (DHT). There is a greater need for knowledge of NP-induced reproductive toxicity since the production of engineered nanoparticles is continuously increasing with the nano revolution; therefore, the risk of exposure to nanoparticles is very common.

## Data Availability

Original data are available on request.

## Supplementary Materials

The following supporting information can be downloaded at https://www.mdpi.com/article/10.3390/nano13111783/s1, Text S1: Evaluation of toxicological endpoints and DanioScope™ analysis; Text S2: Immunohistochemical analysis on zebrafish larvae; Text S3: TiO_2_-NPs accumulation; Text S4: Histological Examination; Text S5: Protocol for preparation of semithin sections and Electron microscopy study; Text S6: RNA extraction and qRT-PCR; Text S7: Crystal structure of sex hormone-binding globulin (SHBG); Figure S1: Coagulated eggs rate; Figure S2: Hatching rate; Figure S3: Body length and beats per minute of larvae.

## References

[1] Wilson N. (2018). Nanoparticles: Environmental problems or problem solvers?. BioScience.

[2] Chiavola A., Amato E.D., Stoller M., Chianese A., Boni M.R. (2016). Application of iron based nanoparticles as adsorbents for arsenic removal from water. Chem. Eng. Trans..

[3] Vilardi G., Ochando-Pulido J.M., Stoller M., Verdone N., Di Palma L. (2018). Fenton oxidation and chromium recovery from tannery wastewater by means of iron-based coated biomass as heterogeneous catalyst in fixed-bed columns. Chem. Eng. J..

[4] Piccinno F., Gottschalk F., Seeger S., Nowack B. (2012). Industrial production quantities and uses of ten engineered nanomaterials in Europe and the world. J. Nanoparticle Res..

[5] Sharma S., Sharma R.K., Gaur K., Cátala Torres J.F., Loza-Rosas S.A., Torres A., Saxena M., Julin M., Tinoco A.D. (2019). Fueling a hot debate on the application of TiO_2_ nanoparticles in sunscreen. Materials.

[6] Tan W., Peralta-Videa J.R., Gardea-Torresdey J.L. (2018). Interaction of titanium dioxide nanoparticles with soil components and plants: Current knowledge and future research needs-A critical review. Environ. Sci. Nano.

[7] Scuderi V., Impellizzeri G., Romano L., Scuderi M., Nicotra G., Bergum K., Irrera A., Svensson B., Privitera V. (2014). TiO_2_-coated nanostructures for dye photo-degradation in water. Nanoscale Res. Lett..

[8] Zimbone M., Cacciato G., Spitaleri L., Egdell R.G., Grimaldi M.G., Gulino A. (2018). Sb-doped titanium oxide: A rationale for its photocatalytic activity for environmental remediation. ACS Omega.

[9] Guo M., Song W., Wang T., Li Y., Wang X., Du X. (2015). Phenyl-functionalization of titanium dioxide-nanosheets coating fabricated on a titanium wire for selective solid-phase microextraction of polycyclic aromatic hydrocarbons from environment water samples. Talanta.

[10] Ohsaka T., Shinozaki K., Tsuruta K., Hirano K. (2008). Photo-electrochemical degradation of some chlorinated organic compounds on n-TiO_2_ electrode. Chemosphere.

[11] Lee Y.S., Kim S.J., Venkateswaran P., Jang J.S., Kim H., Kim J.G. (2008). Anion co-doped Titania for solar photocatalytic degradation of dyes. Carbon Lett..

[12] Kim S.H., Lee S.W., Lee G.M., Lee B.T., Yun S.T., Kim S.O. (2016). Monitoring of TiO_2_-catalytic UV-LED photo-oxidation of cyanide contained in mine wastewater and leachate. Chemosphere.

[13] Nguyen A.T., Hsieh C.T., Juang R.S. (2016). Substituent effects on photodegradation of phenols in binary mixtures by hybrid H_2_O_2_ and TiO_2_ suspensions under UV irradiation. J. Taiwan Inst. Chem. Eng..

[14] Moon G.H., Kim D.H., Kim H.I., Bokare A.D., Choi W. (2014). Platinum-like behavior of reduced graphene oxide as a cocatalyst on TiO_2_ for the efficient photocatalytic oxidation of arsenite. Environ. Sci. Technol. Lett..

[15] Chen Z., Li Y., Guo M., Xu F., Wang P., Du Y., Na P. (2016). One-pot synthesis of Mn-doped TiO_2_ grown on graphene and the mechanism for removal of Cr (VI) and Cr (III). J. Hazard. Mater..

[16] Weir A., Westerhoff P., Fabricius L., Hristovski K., Von Goetz N. (2012). Titanium dioxide nanoparticles in food and personal care products. Environ. Sci. Technol..

[17] Wu F., Hicks A.L. (2020). Estimating human exposure to titanium dioxide from personal care products through a social survey approach. Integr. Environ. Assess. Manag..

[18] Larue C., Castillo-Michel H., Sobanska S., Trcera N., Sorieul S., Cecillon L., Ouerdane L., Legros S., Sarret G. (2014). Fate of pristine TiO_2_ nanoparticles and aged paint-containing TiO_2_ nanoparticles in lettuce crop after foliar exposure. J. Harzard. Mater..

[19] Klaine S.J., Alvarez P.J., Batley G.E., Fernandes T.F., Handy R.D., Lyon D.Y., Mahendra S., McLaughlin M.J., Lead J.R. (2008). Nanomaterials in the environment: Behavior, fate, bioavailability, and effects. Environ. Toxicol. Chem..

[20] Handy R.D., Owen R., Valsami-Jones E. (2008). The ecotoxicology of nanoparticles and nanomaterials: Current status, knowledge gaps, challenges, and future needs. Ecotoxicology.

[21] Wu T., Tang M. (2018). Review of the effects of manufactured nanoparticles on mammalian target organs. J. Appl. Toxicol..

[22] Nemmar A., Hoet P.M., Vanquickenborne B., Dinsdale D., Thomeer M., Hoylaerts M.F., Vanbilloen H., Mortelmans L., Nemery B. (2002). Passage of inhaled particles into the blood circulation in humans. Circulation.

[23] Nel A., Xia T., Madler L., Li N. (2006). Toxic potential of materials at the nanolevel. Science.

[24] Mu Q., Jiang G., Chen L., Zhou H., Fourches D., Tropsha A., Yan B. (2014). Chemical basis of interactions between engineered nanoparticles and biological systems. Chem. Rev..

[25] Sajid M., Ilyas M., Basheer C., Tariq M., Daud M., Baig N., Shehzad F. (2015). Impact of nanoparticles on human and environment: Review of toxicity factors, exposures, control strategies, and future prospects. Environ. Sci. Pollut. Res..

[26] Behzadi S., Serpooshan V., Tao W., Hamaly M.A., Alkawareek M.Y., Dreaden E.C., Brown D., Alkilany A.M., Farokhzad O.C., Mahmoudi M. (2017). Cellular uptake of nanoparticles: Journey inside the cell. Chem. Soc. Rev..

[27] Iavicoli I., Fontana L., Leso V., Bergamaschi A. (2013). The effects of nanomaterials as endocrine disruptors. Int. J. Mol. Sci..

[28] Matthiessen P., Johnson I. (2007). Implications of research on endocrine disruption for the environmental risk assessment, regulation and monitoring of chemicals in the European. Union. Environ. Pollut..

[29] Jeong T.Y., Simpson M.J. (2021). Endocrine disruptor exposure causes infochemical dysregulation and an ecological cascade from zooplankton to algae. Environ. Sci. Technol..

[30] Iavicoli I., Fontana L., Bergamaschi A. (2009). The effects of metals as endocrine disruptors. J. Toxicol. Environ. Health B.

[31] Fang Q., Shi Q., Guo Y., Hua J., Wang X., Zhou B. (2016). Enhanced bioconcentration of bisphenol A in the presence of nano-TiO_2_ can lead to adverse reproductive outcomes in zebrafish. Environ. Sci. Technol..

[32] Kotil T., Akbulut C., Yön N.D. (2017). The effects of titanium dioxide nanoparticles on ultrastructure of zebrafish testis (*Danio rerio*). Micron.

[33] Nüsselin-Volhard C., Dahm R. (2000). Zebrafish a Practial Approch.

[34] Pecoraro R., Marino F., Salvaggio A., Capparucci F., Di Caro G., Iaria C., Salvo A., Rotondo A., Tibullo D., Guerriero G. (2017). Evaluation of Chronic Nanosilver Toxicity to Adult Zebrafish. Front. Physiol..

[35] OECD (2013). Test No. 236: Fish Embryo Acute Toxicity (FET) Test, OECD Guidelines for the Testing of Chemicals.

[36] Sobanska M., Scholz S., Nyman A.M., Cesnaitis R., Gutierrez A.S., Klüver N., De Coen W. (2018). Applicability of the fish embryo acute toxicity (FET) test (OECD 236) in the regulatory context of registration, evaluation, authorisation, and restriction of chemicals (REACH). Environ. Toxicol. Chem..

[37] Pecoraro R., Salvaggio A., Marino F., Di Caro G., Capparucci F., Lombardo B.M., Messina G., Scalisi E.M., Tummino M., Loreto F. (2017). Metallic nano-composite toxicity evaluation by zebrafish embryo toxicity test with identification of specific exposure biomarkers. Curr. Protoc. Toxicol..

[38] Kimmel C.B., Ballard W.W., Kimmel S.R., Ullmann B., Schilling T.F. (1995). Stages of embryonic development of the zebrafish. Dev. Dynam..

[39] Giannaccini M., Cushieri A., Dente L., Raffa V. (2014). Non-mammalian vertebrate embryos as models in nanomedicine. Nanomed. Nanotechnol. Biol. Med..

[40] Zimbone M., Calcagno L., Messina G., Baeri P., Compagnini G. (2011). Dynamic light scattering and UV–vis spectroscopy of gold nanoparticles solution. Mater. Lett..

[41] Zimbone M., Musumeci P., Baeri P., Messina E., Boninelli S., Compagnini G., Calcagno L. (2012). Rotational dynamics of gold nanoparticle chains in water solution. J. Nanoparticle Res..

[42] Kristofco L.A., Haddad S.P., Chambliss C.K., Brooks B.W. (2018). Differential uptake of and sensitivity to diphenhydramine in embryonic and larval zebrafish. Environ. Toxicol. Chem..

[43] Rawson D.M., Zhang T., Kalicharan D., Jogebloed W.L. (2001). Field emission scanning electron microscopy and transmission electron microscopy studies of the chorion, plasma membrane and syncytial layers of the gastrula-stage embryo of the zebrafish *Brachy Danio rerio*: A consideration of the structural and functional relationships with respect to cryoprotectant penetration. Aquacult. Res..

[44] Fent K., Weisbrod C.J., Wirth-Heller A., Pieles U. (2010). Assessment of uptake and toxicity of fluorescent silica nanoparticles in zebrafish (*Danio rerio*) early lifestages. Aquat. Toxicol..

[45] Cheng J., Flahaut E., Cheng S.H. (2007). Effect of carbon nanotubes on developing zebrafish (*Danio rerio*) embryos. Environ. Toxicol. Chem..

[46] Pereira A.C., Gomes T., Machado M.R.F., Rocha T.L. (2019). The zebrafish embryotoxicity test (ZET) for nanotoxicity assessment: From morphological to molecular approach. Environ. Pollut..

[47] Federici G., Shaw B.J., Handy R.D. (2007). Toxicity of titanium dioxide nanoparticles to rainbow trout (*Oncorhynchus mykiss*): Gill injury, oxidative stress, and other physiological effects. Aquat. Toxicol..

[48] Chen T.H., Lin C.Y., Tseng M.C. (2011). Behavioral effects of titanium dioxide nanoparticles on larval zebrafish (Danio rerio). Mar. Pollut. Bull..

[49] Wang Y.J., He Z.Z., Fang Y.W., Xu Y., Chen Y.N., Wang G.Q., Yang Y.Q., Yang Z., Li Y.H. (2014). Effect of titanium dioxide nanoparticles on zebrafish embryos and developing retina. Int. J. Ophthalmol..

[50] Faria M., Navas J.M., Soares A.M., Barata C. (2014). Oxidative stress effects of titanium dioxide nanoparticle aggregates in zebrafish embryos. Sci. Total Environ..

[51] Bai W., Zhang Z., Tian W., He X., Ma Y., Zhao Y., Chai Z. (2010). Toxicity of zinc oxide nanoparticles to zebrafish embryo: A physicochemical study of toxicity mechanism. J. Nanopart. Res..

[52] Johnson A., Carew E., Sloman K.A. (2007). The effects of copper on the morphological and functional development of zebrafish embryos. Aquat. Toxicol..

[53] Asharani P.V., Lianwu Y.I., Gong Z., Valiyaveettil S. (2011). Comparison of the toxicity of silver, gold and platinum nanoparticles in developing zebrafish embryos. Nanotoxicology.

[54] Chakraborty C., Sharma A.R., Sharma G., Lee S.S. (2016). Zebrafish: A complete animal model to enumerate the nanoparticle toxicity. J. Nanobiotechnol..

[55] Cerbinskaite A., Mukhopadhyay A., Plummer E.R., Curtin N.J., Edmondson R.J. (2012). Defective homologous recombination in human cancers. Cancer Treat. Rev..

[56] De Murcia J.M., Niedergang C., Trucco C., Ricoul M., Dutrillaux B., Mark M., Oliver F.J., Masson M., Dierich A., LeMeur M. (1997). Requirement of poly (ADP-ribose) polymerase in recovery from DNA damage in mice and in cells. Proc. Natl. Acad. Sci. USA.

[57] Heacock M.L., Stefanick D.F., Horton J.K., Wilson S.H. (2010). Alkylation DNA damage in combination with PARP inhibition results in formation of S-phase-dependent double-strand breaks. DNA Repair.

[58] Kondo N., Takahashi A., Ono K., Ohnishi T. (2010). DNA damage induced by alkylating agents and repair pathways. J. Nucleic Acids..

[59] Lindquist S., Craig E.A. (1988). The heat-shock proteins. Ann. Rev. Genet..

[60] Hu Y.L., Qi W., Han F., Shao J.Z., Gao J.Q. (2011). Toxicity evaluation of biodegradable chitosan nanoparticles using a zebrafish embryo model. Int. J. Nanomed..

[61] Lin S., Zhao Y., Xia T., Meng H., Ji Z., Liu R., George S., Xiong S., Wang X., Zhang H. (2011). High content screening in zebrafish speeds up hazard ranking of transition metal oxide nanoparticles. ACS Nano.

[62] Dziegiel P. (2004). Expression of metallothioneins in tumor cells. Pol. J. Pathol..

[63] Brundo M.V., Pecoraro R., Marino F., Salvaggio A., Tibullo D., Saccone S., Bramanti V., Buccheri M.A., Impellizzeri G., Scuderi V. (2016). Toxicity evaluation of new engineered nanomaterials in zebrafish. Front. Physiol..

[64] Lin H.Y., Muller Y.A., Hammond G.L. (2010). Molecular and structural basis of steroid hormone binding and release from corticosteroid-binding globulin. Mol. Cell. Endocrinol..

[65] Hammond G.L. (2011). Diverse roles for sex hormone-binding globulin in reproduction. Biol. Reprod..

[66] Miguel-Queralt S., Hammond G.L. (2008). Sex hormone-binding globulin in fish gills is a portal for sex steroids breached by xenobiotics. Endocrinology.

[67] Bobe J., Guiguen Y., Fostier A. (2010). Diversity and biological significance of sexhormone-binding globulin in fish, an evolutionary perspective. Mol. Cell. Endocrinol..

[68] Miguel-Queralt S., Knowlton M., Avvakumov G.V., Al-Nouno R., Kelly G.M., Hammond G.L. (2004). Molecular and functional characterization of sex hormone binding globulin in zebrafish. Endocrinology.

[69] Dechaud H., Ravard C., Claustrat F., de la Perrière A.B., Pugeat M. (1999). Xenoestrogen interaction with human sex hormone-binding globulin (hSHBG). Steroids.

[70] Hodgert Jury H., Zacharewski T.R., Hammond G.L. (2000). Interactions between human plasma sex hormone-binding globulin and xenobiotic ligands. J. Steroid Biochem. Mol. Biol..

[71] Hong H., Branham W.S., Ng H.W., Moland C.L., Dial S.L., Fang H., Perkins R., Sheehan D., Tong W. (2015). Human sex hormone-binding globulin binding affinities of 125 structurally diverse chemicals and comparison with their binding to androgen receptor, estrogen receptor, and α-fetoprotein. Toxicol. Sci..

[72] Desvergne B., Feige J.N., Casals-Casas C. (2009). PPAR-mediated activity of phthalates: A link to the obesity epidemic?. Mol. Cell. Endocrinol..

[73] Chen P., Wang Q., Chen M., Yang J., Wang R., Zhong W., Zhu L., Yang L. (2018). Antagonistic estrogenic effects displayed by bisphenol AF and perfluorooctanoic acid on zebrafish (*Danio rerio*) at an early developmental stage. Environ. Sci. Technol. Lett..

[74] Haritos A.A., Goodall G.J., Horecker B.L. (1984). Prothymosin alpha: Isolation and properties of the major immunoreactive form of thymosin alpha 1 in rat thymus. Proc. Natl. Acad. Sci. USA.

[75] Dosil M., Freire M., Gomez-Marquez J. (1990). Tissue-specific and differential expression of prothymosin alpha gene during rat development. FEBS Lett..

[76] Yoshida S., Ono N., Tsukue N., Oshio S., Umeda T., Takano H., Takeda K. (2006). *In utero* exposure to diesel exhaust increased accessory reproductive gland weight and serum testosterone concentration in male mice. Environ. Sci..

[77] Asare N., Instanesa C., Sandberga W.J., Refsnesa M., Schwarzea P., Kruszewskib M., Brunborg G. (2012). Cytotoxic and genotoxic effects of silver nanoparticles in testicular cells. Toxicology.

[78] Gao G., Ze Y., Zhao X., Sang X., Zheng L., Ze X., Gui S., Sheng L., Sun Q., Hong J. (2013). Titanium dioxide nanoparticle-induced testicular damage, spermatogenesis suppression, and gene expression alterations in male mice. J. Hazard. Mater..

[79] De Jong W.H., Hagens W.I., Krystek P., Burger M.C., Sips A.J., Geertsma R.E. (2008). Particle size-dependent organ distribution of gold nanoparticles after intravenous administration. Biomaterials.

[80] Sadauskas E., Jacobsen N.R., Danscher G., Stoltenberg M., Vogel U., Larsen A., Kreyling W., Wallin H. (2009). Biodistribution of gold nanoparticles in mouse lung following intratracheal instillation. Chem. Cent. J..

[81] Lankveld D.P., Oomen A.G., Krystek P., Neigh A., Troostde Jong A., Noorlander C.W., Van Eijkeren J.C., Geertsma R.E., De Jong W.H. (2010). The kinetics of the tissue distribution of silver nanoparticles of different sizes. Biomaterials.

[82] Lankveld D.P., Rayavarapu R.G., Krystek P., Oomen A.G., Verharen H.W., van Leeuwen T.G., De Jong W.H., Manohar S. (2011). Blood clearance and tissue distribution of PEGylated and non-PEGylated gold nanorods after intravenous administration in rats. Nanomedicine.

[83] Lead J.R., Batley G.E., Alvarez P.J.J., Croteau M.N., Handy R.D., Mclaughlin M.J., Judy J.D., Schrimer K. (2018). Nanomaterials in the environment: Behavior, bioavailability, and effects—An updated review. Environ. Toxicol. Chem..

[84] Griffitt R.J., Lavelle C.M., Kane A.S., Denslow N.D., Barber D.S. (2013). Chronic nanoparticulate silver exposure results in tissue accumulation and transcriptomic changes in zebrafish. Aquat. Toxicol..

[85] Xiong D., Fang T., Yu L., Sima X., Zhu W. (2011). Effects of nano-scale TiO_2_, zno and their bulk counterparts on zebrafish: Acute toxicity, oxidative stress and oxidative damage. Sci. Total Environ..

[86] Griffitt R.J., Hyndman K., Denslow N.D., Barber D.S. (2009). Comparison of molecular and histological changes in zebrafish gills exposed to metallic nanoparticles. Toxicol. Sci..

[87] Song G., Lin L., Liu L., Wang K., Ding Y., Niu Q., Mu L., Wang H., Shen H., Guo S. (2017). Toxic effects of anatase titanium dioxide nanoparticles on spermatogenesis and testicles in male mice. Pol. J. Environ. Stud..

[88] Baker B.B., Yee J.S., Meyer D.N., Yang D., Baker T.R. (2016). Histological and transcriptomic changes in male zebrafish testes due to early life exposure to low level 2, 3, 7, 8-tetrachlorodibenzo-p-dioxin. Zebrafish.

[89] Nittoli V., Colella M., Porciello A., Reale C., Roberto L., Russo F., Russo N.A., Porreca I., De Felice M., Mallardo M. (2021). Multi Species Analyses Reveal Testicular T3 Metabolism and Signalling as a Target of Environmental Pesticides. Cells.

[90] Lora A.J., Molina A.M., Bellido C., Blanco A., Monterde J.G., Moyano M.R. (2016). Adverse effects of bisphenol A on the testicular parenchyma of zebrafish revealed using histomorphological methods. Vet. Med..

[91] Siiteri P.K., Murai J.T., Raymoure W.J., Kuhn R.W., Hammond G.L., Nisker J.A. (1982). The Serum Transport of Steroid Hormones. Proceedings of the 1981 Laurentian Hormone Conference.

[92] Joseph D.R. (1994). Structure, function, and regulation of androgen-binding protein/sex hormone-binding globulin. Vitam. Horm..

[93] Mak P., Callard G.V. (1987). A novel steroid-binding protein in the testis of the dogfish Squalus acanthias. Gen. Comp. Endocrinol..

[94] Ovrevik J., Stenersen J., Nilssen K., Tollefsen K.E. (2001). Partial characterization of a sex steroid-binding protein in plasma from arctic charr (*Salvelinus alpinus* L.). Gen. Comp. Endocrinol..

[95] Milligan S.R., Khan O., Nash M. (1998). Competitive binding of xenobiotic oestrogens to rat fetoprotein and to sex steroid binding proteins in human and rainbow trout (*Oncorhynchus mykiss*) plasma. Gen. Comp. Endocrinol..

[96] Tollefsen K.E. (2002). Interaction of estrogen mimics, singly and in combination, with plasma sex steroid-binding proteins in rainbow trout (*Oncorhynchus mykiss*). Aquat. Toxicol..

[97] Pryce-Hobby A.C., McMaster M.E., Hewitt L.M., Van Der Kraak G. (2003). The effects of pulp mill effluent on the sex steroid binding protein in white sucker (*Catostomus commersoni*) and longnose sucker (*C. catostomus*). Comp. Biochem. Physiol..

[98] Foucher J.L., Le Bail P.Y., Le Gac F. (1992). Influence of hypophysectomy, castration, fasting, and spermiation on SBP concentration in male rainbow trout (*Oncorhynchus mykiss*). Gen. Comp. Endocrinol..

[99] Hobby A.C., Geraghty D.P., Pankhurst N.W. (2000). Differences in binding characteristics of sex steroid binding protein in reproductive and non reproductive female rainbow trout (*Oncorhynchus mykiss*), black bream (*Acanthopagrus butcheri*), and greenback flounder (*Rhombosolea tapirina*). Gen. Comp. Endocrinol..

[100] Bobe J., Mahé S., Nguyen T., Rime H., Vizziano D., Fostier A., Guiguen Y. (2008). A novel, functional, and highly divergent sex hormone-binding globulin that may participate in the local control of ovarian functions in salmonids. Endocrinology.

[101] Miguel-Queralt S., Underhill C., Devlin R.H., Hammond G.L. (2009). Characterization and Measurement of the Plasma α-and β-Sex Hormone-Binding Globulin Paralogs in Salmon. Endocrinology.

[102] Hryb D.J., Nakhla A.M., Kahn S.M., St George J., Levy N.C., Romas N.A., Rosner W. (2002). Sex hormone-binding globulin in the human prostate is locally synthesized and may act as an autocrine/paracrine effector. J. Biol. Chem..

[103] Tokarz J., Möller G., de Angelis M.H., Adamski J. (2013). Zebrafish and steroids: What do we know and what do we need to know?. J. Steroid Biochem. Mol. Biol..

[104] Hering D.M., Olenski K., Kaminski S. (2014). Genome-wide association study for poor sperm motility in Holstein-Friesian bulls. Anim. Reprod. Sci..

[105] Urbatzka R., Watermann B., Lutz I., Kloas W. (2009). Exposure of Xenopus laevis tadpoles to finasteride, an inhibitor of 5-alpha reductase activity, impairs spermatogenesis and alters hypophyseal feedback mechanisms. J. Mol. Endocrinol..

[106] Kang H.J., Imperato-McGinley J., Zhu Y.S., Rosenwaks Z. (2014). The effect of 5α-reductase-2 deficiency on human fertility. Fertil. Steril..

[107] Margiotta-Casaluci L., Hannah R.E., Sumpter J.P. (2013). Mode of action of human pharmaceuticals in fish: The effects of the 5-alpha-reductase inhibitor, dutasteride, on reproduction as a case study. Aquat. Toxicol..

[108] Garcìa-Garcìa M., Sànchez-Hernàndez M., Garcìa-Hernàndez M.P., Garcìa-Ayala A., Chaves-Pozo E. (2017). Role of 5α-dihydrotestosterone in testicular development of gilthead seabream following finasteride administration. J. Steroid Biochem. Mol. Biol..

[109] Garcia T.X., Costa G.M., França L.R., Hofmann M.C. (2014). Sub-acute intravenous administration of silver nanoparticles in male mice alters Leydig cell function and testosterone levels. Reprod. Toxicol..

[110] Adebayo O.A., Akinloye O., Adaramoye O.A. (2018). Cerium oxide nanoparticle elicits oxidative stress, endocrine imbalance and lowers sperm characteristics in testes of balb/c mice. Andrologia.

[111] Goldstone J.V., McArthur A.G., Kubota A., Zanette J., Parente T., Jönsson M.E., Nelson D.R., Stegeman J.J. (2010). Identification and developmental expression of the full complement of Cytochrome P450 genes in Zebrafish. BMC Genom..

[112] Vallyathan V., Shi X. (1997). The role of oxygen free radicals in occupational and environmental lung diseases. Environ. Health Perspect..

[113] Bar-Ilan O., Louis K.M., Yang S.P., Pedersen J.A., Hamers R.J., Peterson R.E., Heideman W. (2012). Titanium dioxide nanoparticles produce phototoxicity in the developing zebrafish. Nanotoxicology.

[114] Fu P.P., Xia Q., Sun X., Yu H. (2012). Phototoxicity and environmental transformation of polycyclic aromatic hydrocarbons (PAHs)-light-induced reactive oxygen species, lipid peroxidation, and DNA damage. J. Environ. Sci. Health Part C Environ. Carcinog. Ecotoxicol. Rev..

[115] Filho D.W. (1996). Fish antioxidant defenses—A comparative approach. Braz. J. Med. Biol. Res..

[116] Pandey S., Parvez S., Sayeed1 I., Haque R., Bin-Hafeez B., Raisuddin S. (2003). Biomarkers of oxidative stress: A comparative study of river Yamuna fish Wallago attu (Bl. & Schn.). Sci. Total Environ..

[117] Zhu X., Zhou J., Cai Z. (2011). The toxicity and oxidative stress of TiO_2_ nanoparticles in marine abalone (*Haliotis diversicolor supertexta*). Mar. Pollut. Bull..

[118] Van der Oost R., Beyer J., Vermeulen N.P. (2003). Fish bioaccumulation and biomarkers in environmental risk assessment: A review. Environ. Toxicol. Pharmacol..

[119] Zhang J., Shen H., Wang X., Wu J., Xue Y. (2004). Effects of chronic exposure of 2, 4-dichlorophenol on the antioxidant system in liver of freshwater fish Carassius auratus. Chemosphere.

[120] Lin T., Zhou D., Dong J., Jiang F., Chen W. (2016). Acute toxicity of dichloroacetonitrile (DCAN), a typical nitrogenous disinfection by-product (N-DBP), on zebrafish (*Danio rerio*). Ecotox. Environ. Safe.

[121] Asakura H., Kitahora T., Ronald R.W., Victor R.P., Sherma Z. (2018). Antioxidants and Polyphenols in Inflammatory Bowel Disease: Ulcerative Colitis and Crohn Disease. Polyphenols: Prevention and Treatment of Human Disease.

[122] Celardo I., Pedersen J.Z., Traversa E., Ghibelli L. (2011). Pharmacological potential of cerium oxide nanoparticles. Nanoscale.

[123] Nash K.M., Ahmed S. (2015). Nanomedicine in the ROS-mediated pathophysiology: Applications and clinical advances. Nanomed. Nanotechnol. Biol. Med..

