# Supplementary material for: Titanium Dioxide Nanoparticles: Effects on Development and Male Reproductive System
Source: Nanomaterials (Basel). 2023 May 31;13(11):1783. doi: 10.3390/nano13111783 (PMC10254871; doi:10.3390/nano13111783)
Supplement: Supplementary file 1 [file nanomaterials-13-01783-s001.zip › nanomaterials-2384741-supplementary.pdf]

Supplementary Materials

Titanium Dioxide Nanoparticles: Effects on Development and Male Reproductive System

Elena Maria Scalisi [1,*], Roberta Pecoraro [1], Antonio Salvaggio [2], Fabiano Capparucci [3], Cosimo Gianluca Fortuna [4], Massimo Zimbone [5], Giuliana Impellizzeri [5] and Maria Violetta Brundo [1]

[1] Department of Biological, Geological and Environmental Science, University of Catania, 95124 Catania, Italy; roberta.pecoraro@unict.it (R.P.); mariavioletta.brundo@unict.it (M.V.B.)
[2] Experimental Zooprophylactic Institute of Sicily "A. Mirri", 90129 Palermo, Italy; antonio.salvaggio@izssicilia.it
[3] Department of Chemical, Biological, Pharmacological and Environmental Science, University of Messina, 98166 Messina, Italy; fcapparucci@unime.it
[4] Department of Chemical Sciences, University of Catania, 95124 Catania, Italy; cg.fortuna@unict.it
[5] CNR-IMM, 95123 Catania, Italy; ; massimo.zimbone@ct.infn.it (M.Z.); giuliana.impellizzeri@ct.infn.it (G.I.)
* Correspondence: elenamaria.scalisi@unict.it; +39-095-7306040

Text S1: Evaluation of toxicological endpoints and DanioScope™ analysis

The embryos were immobilized on dish of agarose and acclimated for at least 3min before to record videos. For the detection of cardiological activity it was necessary record a video and select the heart area above the ventricle. The software apply a changing pixel intensity algorithm to detect changes in pixel density during ventricular contractions, because this changes are correlated directly with cardiac muscle contraction. Then it automatically calculates the number of beats per second (BPS) and beats per minute (BPM). Finally, morphology measurements as body length of larvae was determinated using images uploaded on DanioScope™. After calibration with stage micrometer scale, DanioScope™ has measured these parameters. All measurements were performed independent.

Text S2: Immunohistochemical analysis on zebrafish larvae

At the end of exposure, all larvae (exposed and controls) were fixed in 4% (w/v) paraformaldehyde for 20 min at room temperature. We have applied the following primary antibodies raised in one species (e.g., mouse and rabbit): anti-rabbit-HSP70 (GeneTex, 1:1000), anti-mouse-PARP-1 (Invitrogen, 1:1000), anti-rabbit-SHBG (GeneTex, 2 µg/ml), anti-mouse-PTMA (abcam, 1:100) and anti-mouse-MTs (GeneTex, 1:1000). After fixation, the larvae were washed with Phosphate Buffered Saline (PBS) (pH 7.4, 0.1 M), permeabilized with PBS-Triton X-100 (for 15 min) to improve antibody penetration and then they were incubated with blocking solution of bovine serum albumin (BSA) for 20 min to blocked non-specific antibody binding. It was followed incubation with the primary antibodies. After the primary antibodies incubation was complete, larvae were washed twice (each time for 5 min) in PBS–Tween 20 to remove the excess of primary antibodies. Thus, the secondary antibodies anti-rabbit and anti-mouse (1:1000 dilution) were used, namely TRITC-conjugated anti-rabbit secondary antibodies for HSP70, and SHBG, while FITC-conjugated anti-mouse secondary antibodies for PARP-1, PTMA and MT. Incubation was performed for 1 hour at 4°C in the dark. Finally, the secondary antibodies were remove through washed in PBS–Tween 20 (2 times for 5 min) at room temperature. The larvae were dehydrated in increasing alcohol solutions (70°, 80° and 95°) for 1 min each and air dried. At the end of protocol, larvae were mounted with DAPI (Abcam) and sealed with rubber cement to be examine under fluorescence microscope (NIKON ECLIPSE Ci fluorescence microscope). The images were capture with the NIKON DS-Qi2 camera connected to fluorescence microscope. TRITC-conjugated anti-rabbit secondary antibodies exhibited a red fluorescence, whereas FITC-conjugated anti-mouse secondary antibody exhibited a green fluorescence.

Text S3: TiO_2-NPs accumulation

100 mg of each organ from treated groups, including the control, were accurately weighed into (15 mL) conical tubes, then they followed an alkaline digestion according to Gray and Colleagues (2013) method. Ultrasonic bath was used to break tissue and release the nanoparticles without altering them (30 min at 37 °C), then the samples were left to digest at room temperature to 24h. Next day, the digested solutions were diluted to 50 mL using high purity water 0.22µm filtered (Millipore, Bedford, MA, USA) to 1% tetramethylammonium hydroxide (TMAH) and 0.1% Triton X-100, useful to prevent particle aggregation. Titanium nanoparticle stock solution was prepared from a TiO_2-NPs standard (60 nm TiO_2 Nano Powder, rutile, 99.9%, AEM) purchased from Nanovision (Brugherio, MB, Italy), while Ti ion standard (1000 mg/L, CPAchem) was used for *sp*ICP-MS calibration of dissolved titanium. All digested samples and calibration solutions were sonicated for 30 min before analysis to allow a homogeneous dispersion. TiO_2-NPs were analyzed using ICP-MS NexION® 350D (Perkin Elmer, Waltham, MA, USA) with the Syngistix Nano Application software (Perkin Elmer, Waltham, MA, USA). Thanks to this technique, data on total TiO_2 particles (Ps-Tot) and TiO_2 nanoparticles (NP <100 nm) were acquired. LOD was 1.3×10^3 particles/mL, while LOQ was 2.5×10^3 particles/mL. Referring to the sample weight and digestion volume used, they resulted 2.6×10^5/g and 5.0×10^5/g, respectively. In addition, LOD in size (LODnm) was estimated 35 nm.

Text S4: Histological Examination

The dissected testes, gills, tissues were fixed in newly made 4% formaldehyde (Bio-Optica) in phosphate buffered saline (Sigma Life Science) overnight at room temperature. After washing with phosphate buffered saline (PBS: 0.1 M, pH 7.4) three times of 10 min each one, all tissue were dehydrated in ascending alcohols series (35°, 50°, 70°, 95°, absolute ethanol) for 10 min each one. After, they were clarified in xylene (1h at room temperature) and embedded in paraffin (VWR-Chemicals) 60°C with (Thermofisher Histostar) tissue processor.

Histological sections 5 µm thick were prepared using microtome (Reichert Jung 1150 Autocut) and collected on microscope slide. At least 10 slides of each tissue were collected. The sections were deparaffinized in xylene and stained with Haematoxylin-Eosin (HE) (Bio-Optica).

Text S5: Protocol for preparation of semithin sections and Electron microscopy study

After fixation in glutaraldehyde, the samples are washed in PBS, then second fixation with osmium trethoxide followed (1%) for 45 min. Washing with PBS and dehydration in ascending alcohol (35°-50°-70°-95°-100°), finally propylene oxide. Samples were treated with 1:1 propylene oxide/ resin mixture for 1h on a rotor, after with 2:1 resin mixture/ propylene oxide overnight. Next day, samples were treated with 100% resin mixture for 30 min then embedded in embedding molds. The polymerization of resin was carry out at 60°C for 48hours. Semithin sections (0.85 µm thick) of testicles were cut using an ultramicrotome (Leica Ultracut UCT). They were stained with toluidine blue (Merck) and evaluated under light microscopy (Nikon eclipse E200) connecting with CMOS camera. Semithin sections provide a high-resolution of the morphology section useful to appreciate the structural and morphology of the tissue exanimated.

Instead for transmission electron microscopy (TEM), the samples embedded in resin were cut to 0.085 µm thick. Ultrathin sections were collected on 300 mesh copper-rhodium grids, then contrasted in uranyl acetate and lead citrate. Observations were made with Delong Instruments Schottky field emission LVEM25 Low-Voltage Electron Microscope, operating at 25 kV in bright-field TEM. TEM images were recorded by a Zyla Andor sCMOS Camera equipped with a 2560x2160 pixels sensor.

Text S6: RNA extraction and qRT-PCR

RNA was extracted with MagCore triXact RNA kit according the procedure of production. 2 mg of frozen testis for each experimental groups (1mg/L, 2 mg/L, 4mg/L and

control) was transfer into a RNase-free microcentrifuge tube. It was added 400 µl RB Buffer (containing β-ME) and it was used a micropestle to grind the tissue for few minutes. The samples were incubate at room temperature for 5 min and then they were applied to the top of column (Filter Column Set) provided by kit. Centrifuged for 2 min at 13000 rpm. The supernatant was transferred to Sample Tube, then it and DNase I mixture (200µl) were placed into the correct well of T-Rack of the instrument (MagCore Nucleic Acid Extractor). The extraction of RNA in a final volume of 60µl was carried out about in 60min. The amount of RNA was quantified by Qubit 4 Fluorometer (Invitrogen). Reverse transcription was performed immediately with SuperScript III First-Strand Synthesis SuperMix (Invitrogen) kit, briefly 6µl of total RNA was transcribed by SuperScript III/RNaseOUT Enzyme Mix reverse transcriptase. Then cDNA was amplified by qRT-PCR (QuantStudio 1 Real-Time PCR System, ThermoFisher) using suitable primers listed in Table 1 and the following program: 95°C for 2 min, 40 cycles of 95°C for 15 sec and 60°C for 20 sec, 95°C for 15sec, 60°C for 1 min and 95°C for 15 sec. The ΔΔCt obtained, are used to calculate the fold gene expression values thanks the formula $2^{\wedge -(\Delta\Delta Ct).}$

Table 1. Listed of Forward (F) and reverse (R) primers used in the gene expression evaluation by RT-qPCR.

Gene	Primers Sequences (5'-3')
SHBG	Forward: GTGCTTTCACTGCGTGATGGC<br>Reverse: TCCCAGGGGGTGCTGAG
SRD5A2	Forward: GCGTACGGACGCTATGTGGA<br>Reverse: GCCTGGCAAACTTTCCGTTG
SOD2	Forward: TGGCCAAGGGTGATGTGACAA<br>Reverse: CACCGCCATTGGGTGACAGA
GPX4B	Forward: TGCAACCAGTTCGGAAAGCA<br>Reverse: GAGCTGCGTCTCCGTTCACA
β-Actina	Forward: GCTGTTTTCCCCTCCATTGTT<br>Reverse: TCCCATGCCAACCATCACT

Text S7: Crystal structure of sex hormone-binding globulin (SHBG)

FLAP, is a software developed by the University of Perugia in collaboration with Pfizer, which describes small molecules and protein structures by analyzing their fingerprints. In particular, the software detects which are the interaction fields (MIFs), calculated in GRID, which represent the interactions between small molecules and defined regions of protein structures (pockets).

Thanks the software function of Structure Based Virtual Screening (SBVS), FLAP can identify the pharmacophores in common between the small molecules under examination and the crystallized protein by focusing attention on a specific active site (pocket).

SHBG is a homodimeric glycoprotein and each monomer consists of a repeat pair of a G-Like (LG) domain. The pocket highlighted by the software is only one and corresponds to the DHT binding area, i.e. between the two Beta sheets (Figure 1).

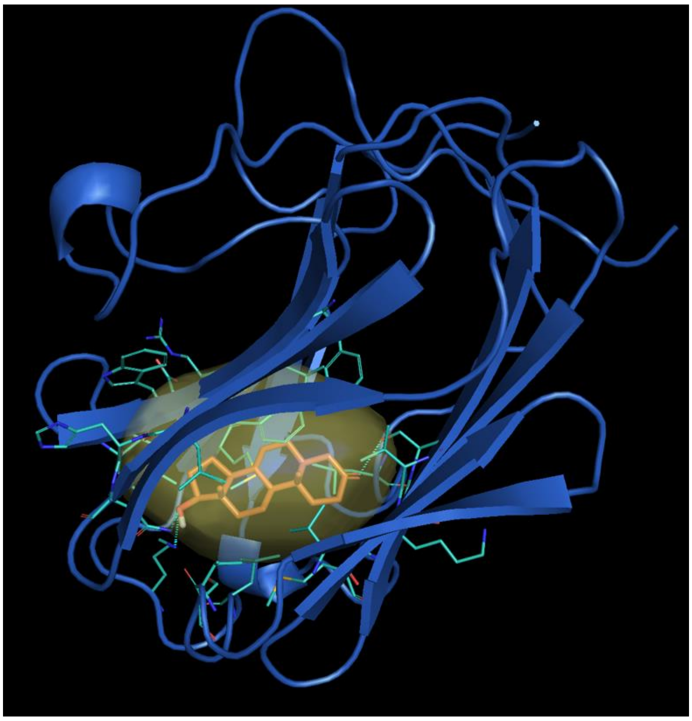

The pocket is quite large in fact there are more amino acid residues involved. The amino acid residues are:

- THR 40
- SER 41
- SER 42
- PHE 56
- GLY 58
- ASP 59
- THR 60
- ASP 65
- TRP 66
- PHE 67
- LEU 80
- HIS 81
- ASN 82
- VAL 105
- LYS 106
- MET 107
- VAL 112
- VAL 127
- SER 128
- LEU 131
- LYS 134
- MET 139
- ARG 140
- ILE 141
- LEU 171

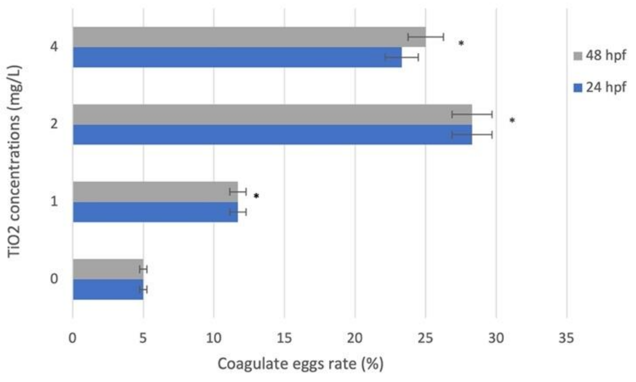

Figure S1. Coagulated eggs rate of embryos exposed to TiO2-NPs ($p<0.05$).

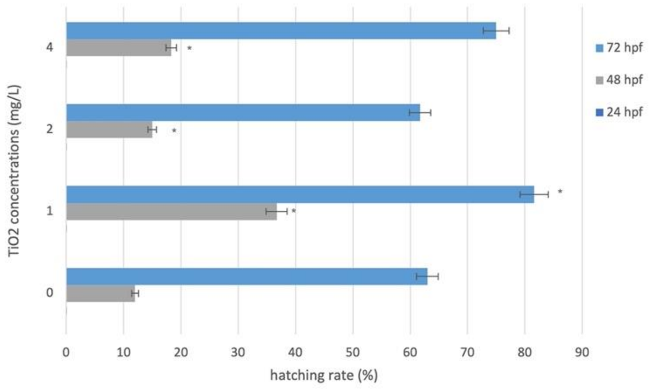

Figure S2. Hatching rate of embryos exposed to TiO2-NPs ($p<0.05$).

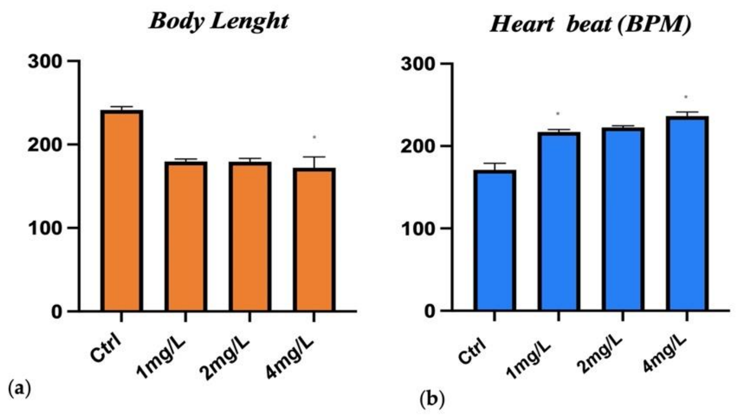

Figure S3. (**a**) Body length and (**b**) beats per minute (BPM) of larvae.
